# Hybrid multi-model framework with knowledge distillation for precise and efficient sickle cell detection

**DOI:** 10.3389/fdgth.2026.1938284

**Published:** 2026-09-29

**Authors:** Metta Manikanta Karthikeya Reddy, Cherukuri Krishna Lathvik, Arumuga Arun R, Karthik K

**Affiliations:** School of Computer Science and Engineering, Vellore Institute of Technology, Vellore, Tamil Nadu, India

**Keywords:** computer vision, edge deployment, hybrid models, knowledge distillation, sickle cell detection

## Abstract

Accurate and timely detection of sickle red blood cells has a significant impact on the patients’ treatment. Developing a computer vision system that is both lightweight and resource-efficient is important for enabling real-time edge-based applications in resource-constrained healthcare environments. This study proposes a hybrid sickle cell detection framework that uses Bi-lightweight Knowledge Distillation (KD)-guided CNNs, combining two lightweight student models from different teacher networks with Machine Learning (ML) classifiers to identify sickle cells with accuracy and efficiency. Using depthwise separable convolution layers, lightweight models are created that drastically lower model complexity. These lightweight models are improved by extracting knowledge from top-performing teacher networks, such as MobileNetV2 and DenseNet121. Using this Knowledge Distillation learning technique, lightweight models can enhance their representational capabilities without adding to their computational complexity. To improve decision-making performance, the Bi-distilled student models are combined with conventional machine learning KNN classifiers to create a hybrid ensemble that combines the advantages of ML-based classification with dual KD-guided CNN-based feature extraction. The Sickle Cell Disease Dataset is used to train and validate the proposed system. The learned student models are then converted into TensorFlow Lite (TFLite) format using Float32 quantization to further reduce computational complexity. In combination with dual KD-guided CNN models and a KNN classifier, the proposed hybrid model achieves a high classification accuracy of 95.45% and an F1-score of 95.02% while being small at only 3.96 MB. Deploying and evaluating the framework on a resource-constrained Raspberry Pi 4 Model B demonstrate its potential for real-time, edge-based, and point-of-care applications.

## 1 Introduction

Sickle cell disease (SCD) is a hereditary blood disorder that affects millions of people around the world. It can lead to a wide range of serious complications, some of which can be fatal, such as severe pain crises, chronic anemia, a higher risk of infections, slow growth in children, organ damage, acute chest syndrome, and strokes (1, 2). For people affected by it, these problems sharply reduce life expectancy and quality of life, especially in poor conditions with a lack of access to the most up-to-date medical facilities (3).

Successful detection of sickle cell disease depends greatly on its timely identification and proper classification. However, traditionally used methods, such as hemoglobin electrophoresis, microscopy of blood smears, and solubility test, being very accurate, cannot be easily scaled because of the need to invest a lot of resources and effort in them. While modern techniques, such as HPLC and genetic tests, are extremely precise, they demand a great financial investment. However, there is a factor of error in the manual process that may make it inconsistent (4). Therefore, it is necessary to look for efficient automated techniques for sickle cell detection. Recently, deep learning solutions have shown themselves to be a promising replacement for traditional techniques due to their high effectiveness and accuracy (47).

One of the efficient methods of automated detection of sickle cell disease includes using Convolutional Neural Networks (CNN). Contrary to classic feature extraction in image recognition tasks that depends on selecting features manually in machine learning algorithms, CNN can learn a hierarchy of features itself, thus providing a better classification. CNN can help develop an efficient automated process by analyzing blood smears and distinguishing sickle-shaped cells from regular erythrocytes (5).

Deep learning algorithms can significantly benefit from transfer learning and pre-trained models. With this transfer learning, it is possible to train a model more efficiently due to the knowledge gained by processing big data volumes. Highly effective feature extraction is available to pre-trained models such as ResNet50, MobileNetV2, Densenet121, and InceptionV3. Thus, there will be no need for a huge number of labeled datasets.

In terms of the implementation of deep learning techniques for real-time detection, computing efficiency becomes a critical factor. One needs to develop a lightweight model that can work on edge devices. The Depth-wise Separable Convolution (DSC) technique can help achieve this goal by reducing the number of parameters and the computational complexity of the network without losing performance (6). Network architectures based on DSC, including MobileNet, provide exceptional effectiveness for medical imaging.

The most effective way of improving the accuracy of a lightweight deep learning model without increasing its computational complexity is the application of the Knowledge Distillation (KD) method. Applying KD allows ensuring the high classification performance of small models by making them use the knowledge gained by more complex teacher networks, such as ResNet, DenseNet, and Inception families, and transfer it to small yet feature-extracting models. Combining KD with DSC ensures an efficient performance-computational complexity trade-off (7).

The recent breakthroughs in medical image analysis show that the incorporation of hybrid approaches, involving both deep learning and machine learning, may be effectively used to achieve the balance between classification performance and computational efficiency. The deep CNN architectures such as ResNet50, DenseNet121, InceptionV3, and MobileNetV2, are powerful in feature extraction and recognition of microscopic images of blood smears. Nevertheless, their straightforward application may lead to limitations in terms of computational resources and the need for a substantial amount of memory. On the contrary, machine learning classifiers, including K-Nearest Neighbors (KNN), SVM, and XGBoost, can use CNN extracted features effectively for the purpose of reliable classification. Thus, the hybrid model combining both CNN-based feature extraction and ML classifier makes use of the benefits provided by both approaches. This will ensure improved generalization and reduced overfitting, as well as increased efficiency in sickle cell detection.

In order to further increase the efficiency of the system, the Bi-lightweight Knowledge Distillation (KD) scheme is introduced. According to the suggested framework, two light-weighted students CNNs are trained independently with the guidance of two efficient teachers from ResNet50, DenseNet121, InceptionV3, and MobileNetV2 networks. Due to the dual KD approach, both student CNNs are able to learn additional discriminative features without compromising the architecture size. Extracted features obtained from training student models are combined and utilized further in machine learning classifiers. Thanks to the combination of the knowledge gained from various teachers with machine learning-based classification, accurate and efficient sickle cell diagnosis can be achieved.

A synergistic framework for constructing an effective system for the prediction of sickle cell disease, involving CNN, transfer learning, DSC approach, knowledge distillation, and machine learning classifiers, is developed. The use of CNN enables efficient feature extraction, while transfer learning enhances generalization. Furthermore, depth-wise separable convolutions significantly reduce computational load, and knowledge distillation results in lightweight yet accurate models without adding any computational complexity. Overall, this combined approach overcomes the drawbacks of conventional techniques and provides reliable sickle cell detection that can be easily scaled.

Diagnosis with the help of deep learning techniques considerably improves the ability to detect sickle cell disease at an earlier stage, which is beneficial not only for patients but also reduces the healthcare cost. Faster clinical decisions and timely action are possible thanks to the use of artificial intelligence for the analysis of blood smear images. The main achievements of this study are outlined below.

1. Using the Depth-wise Separable Convolution technique, the work introduces lightweight CNN models for detecting sickle cells, which are adapted to be implemented in limited-resource settings. With the help of knowledge distillation, proposed lightweight student models are taught to recognize discriminative features in blood smear images by using complex and capacity-reserving models, such as ResNet50, MobileNet, DenseNet121, and InceptionV3, etc.
2. The most informative features of sickle cells are obtained by several student models, where each of them has its unique high-capacity and complex teacher models. As a result, more informative features are extracted, which contribute to accurate sickle cell detection.
3. A hybrid diagnostic scheme is applied to improve generalization, robustness, and sickle cell detection overall. The scheme involves the utilization of features obtained from multi-KD trained CNN models and a conventional ML KNN classifier.

## 2 Literature survey

The following literature review gives insight into prior research work done in this field, is organized into three aspects. To begin with, current literature pertaining to SCD diagnosis using an image-based technique is discussed. Next, the approaches involving the design of lightweight models specifically for the purpose of diagnosing SCD are mentioned. Finally, the application of hybrid learning and ensemble learning approaches is described in detail.

### 2.1 Existing works based on sickle cell detection

Recent advances have been made in the area of utilizing machine learning techniques for SCD diagnosis. Tushabe et al. (2024) proposed an image-based machine learning algorithm, where the features of RBCs are employed in identifying SCD. Though being a non-invasive process, it is relatively unstable in case of low illumination images and images under different stainings (8). It remains unclear how diverse the database in question is in terms of the different protocols, types of microscopes, and patient samples.

Nigan et al. (2024) designed a multi-class model with the ability to detect SCD, elliptocytosis, and schizocytosis using morphological characteristics of the sample, making it more versatile; however, further investigation on its implementation in clinical practice is required (9). It is unclear what the quantitative results in terms of accuracy, sensitivity, or specificity for each of the three diseases (Sickle Cell, Elliptocytosis, Schizocytosis) are. Machado et al. (2024) completed a systematic literature review on machine learning approaches in the context of analyzing SCD data and found that ensemble methods offer higher accuracy rates; nevertheless, the lack of benchmarking datasets is an issue (10). Roy et al. (2024) created a machine learning model based on spectral data, allowing for predicting SCD's severity; however, it is dependent on the presence of spectroscopic equipment (11).

Jennifer et al. (2023) were involved in acquiring and pre-processing blood smear images, potentially segmenting individual cells or analyzing whole fields of view, and then training a CNN to learn hierarchical features and perform classification (12). Liang et al. (2024) proposed a “Patient/Provider Match Tool.” This suggests a system or algorithm that uses data about patients (e.g., disease severity, specific complications, geographical location, preferences) and providers (e.g., specialization, location, availability, experience with SCD) to generate optimal matches. While it enhances coordination and continuity of care, its integration into existing clinical infrastructures and workflows remains a challenge.

### 2.2 Existing works based on building lightweight models for medical applications and sickle cell detection tasks

Goswami et al. (2024) utilized deep learning and explainable artificial intelligence (XAI) to develop an explainable SCD detection method (13). The XAI methods on a DNN that is trained to enable the visualization of the regions in the input image that contributed significantly to the model's prediction, and also improves clinicians’ trust in the output. However, their model was computationally heavy and resource-intensive, which made the integration in portable diagnostic tools difficult. Olatunji et al. (2024) created machine learning algorithms based on structured clinical data to preemptively detect SCD; the approach offers simplicity and speed (14). On the other hand, the approach has a limitation because of the inability to process image-based data, as well as the need to validate the causality between clinical features and SCA occurrence or progression from the medical perspective.

Alotaibi and El-Bathy (2024) created a privacy-preserving deep learning framework using encryption techniques to make the SCD detection process safe and private (15). The framework described the steps that involve encrypting data, performing operations on it securely (such as training a model on encrypted data or transferring encrypted gradients), and obtaining the result without accessing the plaintext data. Despite enhancing patient data protection, the cryptographic layers led to increased processing latency. Subhaga and TP (2025) developed a fast, deep learning-based SCD detection system optimized for efficiency on low-end devices. However, comparisons with other lightweight architectures were not provided (16).

### 2.3 Existing hybrid and ensemble learning approaches in medical applications and sickle cell detection tasks

Jain et al. (2024) proposed a deep learning model combining ResNet with Random Forest to enable classification (17). In spite of its high classification performance, the model is computationally demanding and hard to implement for real-time use in less resource-rich settings. As a result of the high level of accuracy, the classification process seems to be efficient, while other metrics such as precision, recall, and F1-score do not show any breakdown. Deo et al. (2024) proposed a hybrid model between CNN and LSTM used to extract both spatial and sequential features from blood cell images thus improving classification accuracy (18). Hybrid models, including CNN and LSTM (spatial feature learning and sequence understanding, respectively) may be useful in some image analysis cases as they allow capturing of more complex patterns when compared to just a CNN alone. However, the combination of two models makes training complex as well as resource-intensive.

Cardoso et al. (2024) proposed the use of a fusion model that combined the segmented image data with CNN and traditional classifiers to improve detection (6). SCD classification was enhanced by this fusion model, which incorporated information from conventional classifiers, segmented images, and a CNN. This is clearly a multi-modal or ensemble approach since it incorporates different models and features together in order to obtain better results. The improvement in classification accuracy comes at the expense of additional dependency on the segmentation preprocessing steps, which may not be easily implemented in real-time systems.

Alzubaidi et al. (2024) developed a regularized Random Forest model to predict SCD complications using patient records and thus provide interpretability through feature importance (19). This model aims to use machine learning to predict the likelihood of SCD complications and hence proactively manage the condition. Although the generalizability of this model in relation to other datasets is not clear. Dash et al. (2024) conducted a statistical and predictive analysis of hematological parameters of SCD patients to allow early diagnosis through minimum input data (20). This method aimed at improving the diagnosis of SCD through a combination of statistical and predictive analysis approaches. It means that their approach helped in improving the ability to diagnose the disease or the understanding of the role of haematological parameters in diagnosis, although the sensitivity of their method to noisy lab data makes it less efficient. Pal et al. (2024) examined how deep learning can be integrated with genetic data to identify hereditary risks of SCD. It means that one of the benefits of multimodal learning is illustrated, although the limitation is the unavailability of patient genetic data (21).

According to Alotaibi (2025), stacking and bagging are two of the most efficient ways to boost model robustness (15). However, it is impossible to apply such methods in memory-limited systems due to the complexity growth. The research by Abdul et al. (2024) (5) utilized digital morphology for the extraction of detailed features of cells in order to provide better results for detecting SCD. Despite excellent results, the algorithm is dependent on high-resolution images, making it not suitable for use with lower-end microscopes that can be found in rural or remote hospitals.

In summary, today's algorithms emphasize accuracy and feature representation with deep and hybrid models. Still, there are some drawbacks, like computational costs, a lack of standardization, and the impossibility of applying methods to real-life situations. That makes the need for developing lightweight and low-cost models is the major motive of this research work. Comparison of different works from the literature is provided in Table 1 below.

**Table 1:** Comparative analysis of proposed model with other state-of-the-Art models.

Author, Year, and Reference	Key contributions	Merits	Limitations
Jain et al., 2024 (17)	Hybrid ResNet feature extraction with Random Forest classification.	Improves generalization through transfer learning.	Architecture details and evaluation metrics are limited.
Tushabe et al., 2024 (22)	Image-based SCD detection using image processing and classification.	Enables automated analysis of blood smear images.	Quantitative performance metrics are not reported.
Nigan et al., 2024 (9)	Automatic detection of multiple RBC abnormalities.	Supports differentiation of multiple cell morphologies.	Lacks clinical validation and expert comparison.
Deo et al., 2024 (18)	Hybrid CNN-LSTM model for SCA diagnosis.	Achieves high classification performance.	Limited interpretability due to the black-box nature.
Roy et al., 2024 (11)	Spectroscopy-based SCD severity prediction.	Introduces a novel data source for severity assessment.	Clinical validation of severity predictions is absent.
Jennifer et al., 2023 (12)	CNN-based automated SCD detection.	Demonstrates promising classification accuracy.	Not optimized for resource-constrained deployment.
Liang et al., 2024 (23)	ML-based patient-provider matching for SCD care.	Improves access to specialized healthcare.	Matching criteria can be subjective and complex.
Goswami et al., 2024 (6)	XAI-enabled deep learning for SCD detection.	Enhances model interpretability.	Real-world clinical usability remains unexplored.
Olatunji et al., 2024 (14)	ML-based preemptive diagnosis using clinical data.	Utilizes readily available clinical information.	Performance depends heavily on data quality.
Cardoso et al., 2024 (6)	Fusion of CNN and conventional classifiers.	Exploits complementary strengths of multiple models.	Fusion strategy optimization is challenging.
Alotaibi & El-Bathy, 2024 (24)	Privacy-preserving SCD detection framework.	Ensures secure AI-assisted diagnosis.	Integration of cryptographic methods is insufficiently detailed.
Subhaga & TP, 2025 (16)	CNN-based classification of blood smear images.	Enables rapid diagnostic predictions.	Limited model explainability.
Alzubaidi et al., 2024 (19)	Complication prediction using Regularized Random Forest.	Identifies influential clinical risk factors.	Predicting future complications remains challenging.
Dash et al., 2024 (20)	Statistical and predictive analysis of hematological parameters.	Provides data-driven diagnostic insights.	Hematological features alone may be insufficient.
Abdul et al., 2024 (5)	Morphology-based sickle cell identification.	Offers objective and consistent diagnosis.	Highly dependent on dataset quality and diversity.

## 3 Materials and methods

The main objective of this study is to create an efficient sickle cell detector that will be deployed even in low-resource scenarios using Depth-wise Separable Convolution methods to achieve computational efficiency. The knowledge distillation technique helps to optimize the performance of lightweight models. This is because the high-capacity teacher models will help the student models learn powerful representations due to their superior feature extraction abilities. Finally, the knowledge learned by various student models will be combined to design a comprehensive framework capable of detecting diverse morphological changes in the sickle cell. Figure 1 provides a schematic diagram representing the structure of the sickle cell detection system.

**Figure 1:**
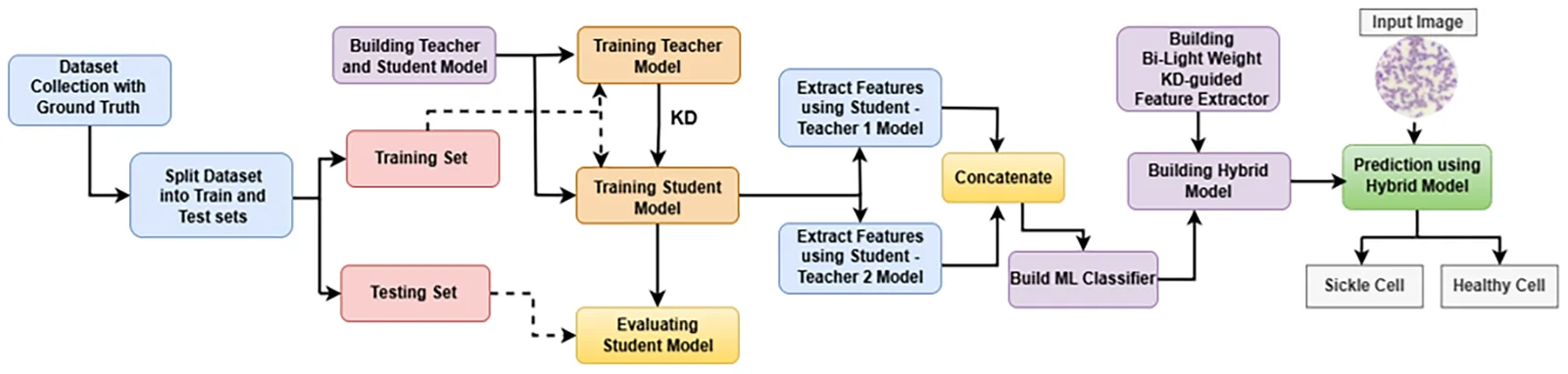
The process pipeline diagram of the proposed approach.

### 3.1 Collecting and pre-processing the dataset

The study utilized a dataset named “Sickle Cell Disease Dataset,” which was prepared by Florence Tushabe, contains clinical and laboratory data related to patients with sickle cell disease, publicly available in the Kaggle repository (22). A couple of sample images from the positive and negative classes are shown in Figure 2. This dataset comprises 422 Diseased (Positive) blood cell images and 147 Healthy (Negative) blood cell images. For training and testing the proposed model, the individual classes present in the dataset are divided in an 8:2 ratio for building the training and testing datasets.

**Figure 2:**
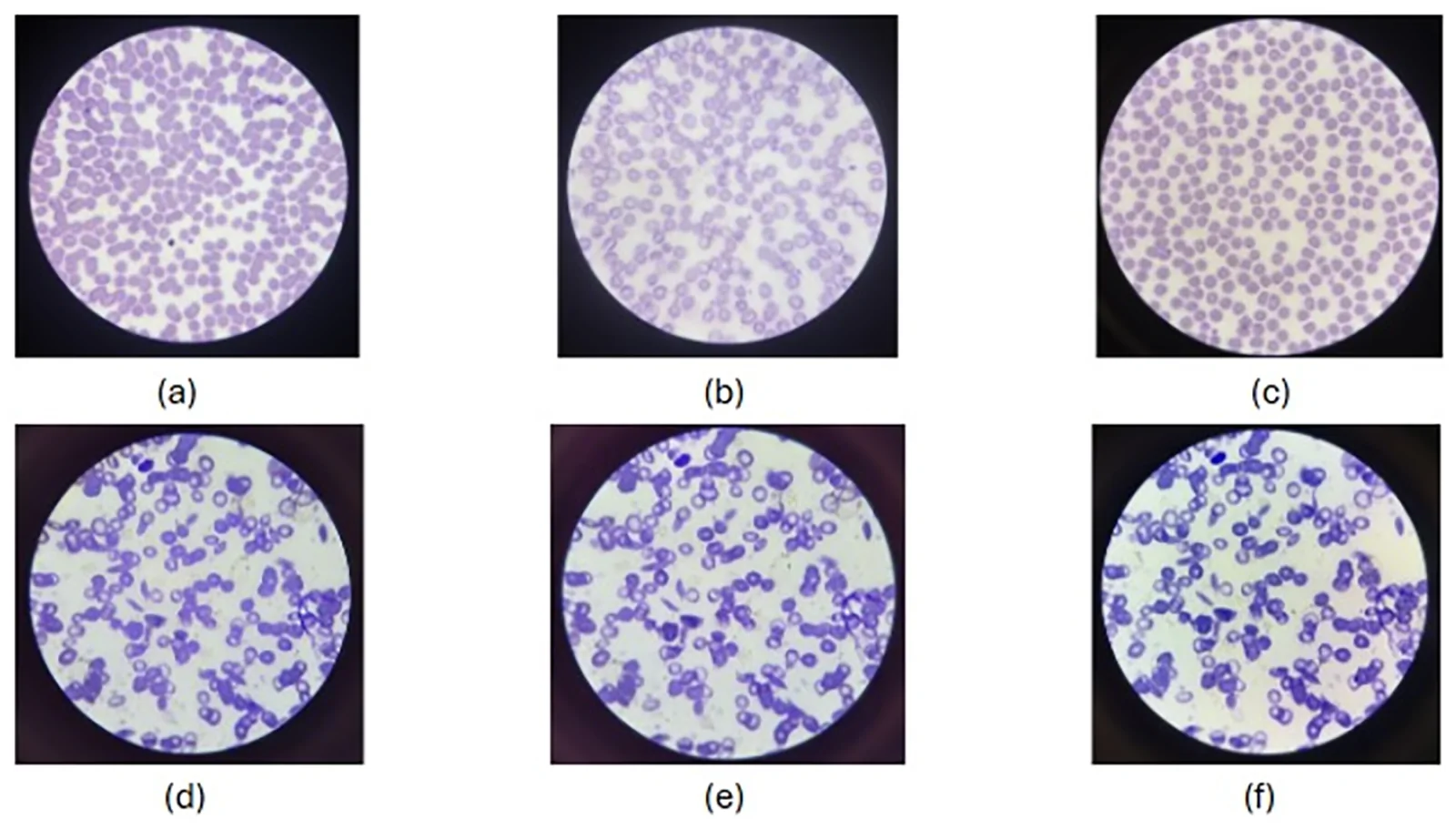
The sample images from the dataset utilized in this study, **(a,b)** healthy (negative) blood cell images, **(d–f)** diseased (positive) blood cell images.

Data augmentation techniques are used in both the training and testing datasets to increase the diversity and robustness to offset the dataset's small size and class imbalance. In order to avoid deformations in the morphology of both sickle cell blood and normal blood cells, the technique of non-deformation enhancement is applied in this experiment; it is an important requirement in the feature extraction and classification process. Non-deformation techniques such as rotations, transpositions, and enhancements in brightness, saturation, sharpness, and contrast are used in order to maintain the structure of blood cells without any distortions in shape. To help the model learn more broadly applicable patterns without depending on fine-grained noise, low-radius Gaussian Blur filtering is also used to simulate imaging irregularities like minute focus changes. To guarantee that the morphological integrity of sickle and healthy red blood cells is maintained, every parameter for these augmentation procedures was carefully chosen. Together, these augmentation techniques increase the size of the effective dataset, decrease overfitting, and strengthen the robustness of the model while maintaining important diagnostic features during training. The effect of these operations on a sample image from diseased and healthy blood cell images is shown in Figure 3. The number of images present in the dataset before and after augmentation operations is recorded in Table 2.

**Figure 3:**
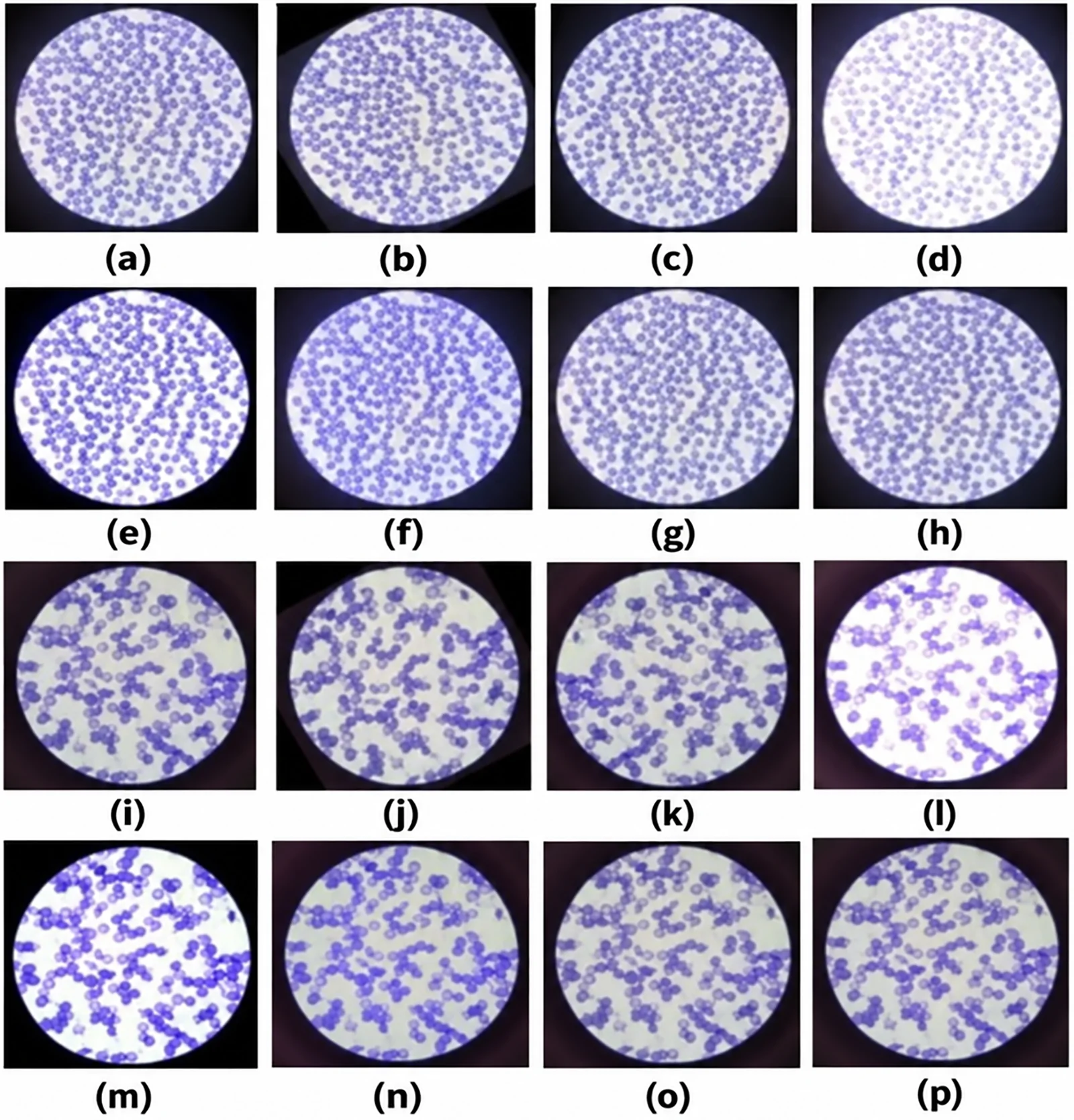
Effect of these non-deformation augmentation operations on a sample image from healthy and diseased blood cell images. **(a,i)** Original Images of healthy and diseased cell images, **(b,j)** Outcome of rotation operation on the healthy and diseased cell images, **(c,k)** Outcome of transposition on the healthy and diseased cell images, **(d,l)** Outcome of brightness adjustment operation on the healthy and diseased cell images, **(e,m)** Outcome of contrast enhancements operation on the healthy and diseased cell images, **(f,n)** Outcome of saturation adjustment operation on the healthy and diseased cell images, **(g,o)** Outcome of sharpness adjustment operation on the healthy and diseased cell images, **(h,p)** Outcome of applying a low-radius Gaussian blur filter operation on the healthy and diseased cell images.

**Table 2:** The details dataset instances before and after augmentation operations.

S.No	Class	Before augmentation			After augmentation	
		Total	Training	Testing	Training	Testing
1	Diseased Blood Cell	422	338	84	800	160
2	Healthy Blood Cell	147	117	30	800	160
Total		569	455	114	1600	320

### 3.2 Building the underlying CNN-based deep learning models used in the knowledge distillation

In a Knowledge Distillation (KD) paradigm, a smaller, more effective model called the student is trained under the guidance of a larger, high-performing deep learning model called the teacher. The student model is small and designed to be deployed in contexts with limited resources, whereas the teacher model is usually a sophisticated CNN architecture that can extract rich and discriminative features.

#### 3.2.1 Teachers models

Teacher models are essential in the Knowledge Distillation (KD) process because they are high-capacity, well-generalized networks that can extract rich and discriminative features from complex input data. Typically, these models are deep and computationally intensive Convolutional Neural Networks (CNNs) that have been trained to achieve high accuracy on the target task, which in this case is classifying sickle and healthy red blood cells from microscopy images.

A teacher model needs to perform exceptionally well and have high generalization skills to offer trustworthy direction in the Knowledge Distillation process. Effective teachers can identify fine-grained morphological characteristics that differentiate sickle-shaped cells from normal ones, in addition to outperforming students in classification accuracy. As a result, the student model's classification performance is eventually improved by learning more complex and discriminative feature representations.

The well-known CNN models ResNet50, DenseNet121, InceptionV3, and MobileNetV2 have been chosen for our work as teacher models due to their established feature extraction capacity in different existing image recognition problems (25–28). Although the models mentioned above are trained using the weights pre-trained on the ImageNet dataset, they play a significant role in the process of detecting sickle cells, since they already have some understanding of general visual features, which can be further tuned to distinguish specific morphological differences between sickle and normal red blood cells. It becomes possible to achieve faster convergence, better accuracy, and greater generalization, which is important for small, imbalanced medical datasets. As teacher models, they provide good guidance to the student models during the Knowledge Distillation procedure.

##### 3.2.1.1 ResNet50

ResNet50 is a deep convolutional neural network that successfully tackles the degradation issue that frequently arises in very deep neural networks by introducing residual learning. The accuracy of traditional models often decreases as network depth increases, not due to overfitting but rather due to optimization challenges. This is addressed by ResNet50 by the use of skip connections, which preserve information and mitigate vanishing gradient problems by allowing gradients to flow through the identity mappings (21, 29). Equation 1 defines the residual mapping that each Residual Block in ResNet learns, and Equation 2 lists the Residual Block's output. This approach speeds up convergence and simplifies optimization by allowing the model to learn only the difference between the input and the output.

F(x)=H(x)−x (1)

Y=F(x)+x (2)

where, x – Input for the block, H(x) - Underlying desired mapping, F(x) – Residual function, Y – Output of residual block.

The ResNet50 model has 50 layers, which include convolution and max-pooling layers. There are then 16 residual blocks arranged in 4 stages (12). To preserve high representational power while reducing computing cost, each block uses a bottleneck architecture with 1  ×  1, 3 × 3, and 1 × 1 convolutional layers. This network architecture is completed with a global average pooling layer followed by a fully connected layer. The structure of the ResNet50 architecture is visualized in Figure 4. The model's deep and well-structured design enables it to extract robust, hierarchical features, making it ideal for use as a teacher model in the sickle cell classification knowledge distillation process. In this role, it can effectively guide lightweight student networks.

**Figure 4:**
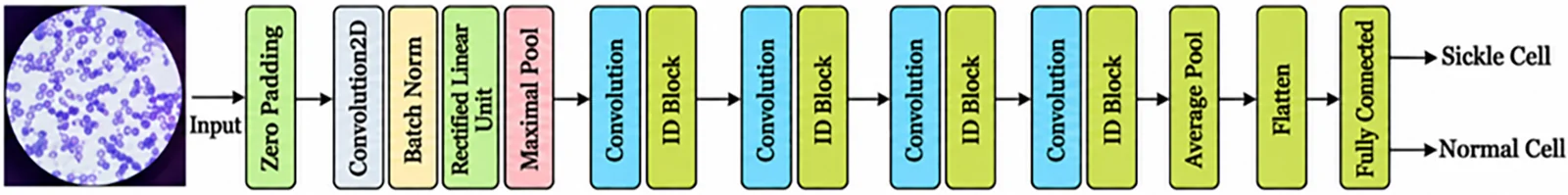
The structure of the ResNet50 model.

##### 3.2.1.2 MobilenetV2

MobileNetV2 is the architecture of a Convolutional Neural Network which is efficient and lightweight in terms of resource requirements but still maintains its accuracy. It has two key architectural innovations: Linear Bottleneck and Inverted Residual Blocks that together lead to an increase in representational capacity and efficiency.

Inverted Residual Blocks of MobileNetV2 include three main stages: Projection, Depthwise Convolution, and Expansion. At the first stage of Expansion, low-dimensional input data are transformed into high-dimensional ones by means of 1 × 1 pointwise convolution (Equation 3). Then, in the Depthwise Convolution stage, independent filtering of every individual channel is done with the help of spatial filter (Equation 4). Finally, in the Projection stage, linear 1 × 1 convolution (Equation 5) is used to decrease the number of dimensions of the feature maps down to original ones. The key feature of this stage is the absence of non-linear activation, which leads to the creation of a Linear Bottleneck and thus allows for preserving of information in low-dimensional spaces. If the input and output dimensionality coincide, then the skip connection is applied (Equation 6). Thus, it facilitates backpropagation of gradients and reuse of features. Architecture of MobileNetV2 is visualized in Figure 5.

$$X_{EP}=ReLU6(W_{EP}\times X)$$ (3)

$$X_{DW\;}=RELU6\left(W_{DW}\;e\;X_{EP}\right)$$ (4)

$$X_{OP}=\;W_{P}\times \;X_{DW}\;\;$$ (5)

$$Y=X+\;X_{OP}$$ (6)

where X – Input tensor, $W_{EP}$, $W_{DW}$, $W_{PR}$ – Weight Matrices for the expansion, depth-wise, and projection layers, respectively, e - Channel-wise depth-wise convolution, RELU6 – ReLU6 is the activation function used to introduce non-linearity while capping outputs at 6, $X_{EP}$, $X_{DW}$, $X_{PR}$ – Outcome of the expansion, depth-wise, and projection layers, respectively, y – outcome of the skip connection.

**Figure 5:**
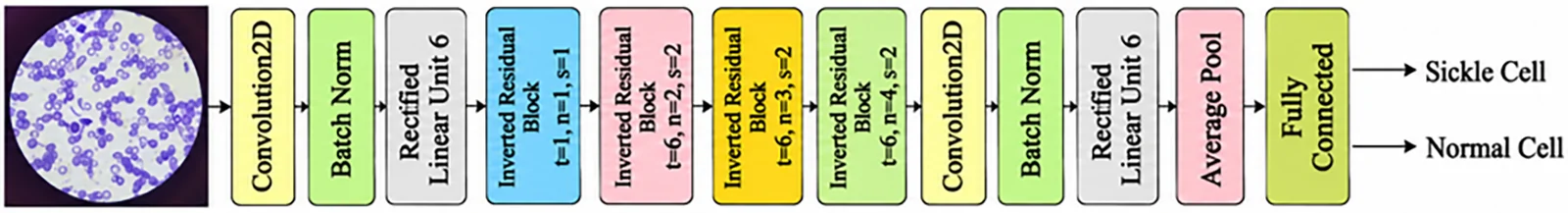
The structure of the MobileNetV2 model.

MobileNetV2's effective usage of depthwise separable convolutions and high-dimensional intermediate representations through Inverted Residual Blocks and Linear Bottlenecks allows it to perform exceptionally well in feature extraction despite its small size. It is ideally suited as a teacher model in Knowledge Distillation (KD) because of its design, which allows it to retain high efficiency while capturing rich features. It helps student models learn the subtle morphological differences between sickle and healthy red blood cells by efficiently transferring discriminative traits to them.

##### 3.2.1.3 InceptionV3

InceptionV3 is an advanced CNN model developed by Google and represents a good balance between the high level of accuracy and low level of computational cost (30). In terms of its superiority to other models, Inception V3 uses architectural innovations that allow for maximum learning at minimal additional computation cost. These include regularizations, label smoothing, auxiliary classifiers, and factorized convolutions.

Factorized convolutions use the concept of decomposing computationally intensive operations, such as 5 × 5 convolutions, into multiple less computationally intensive 3 × 3 or asymmetric operations, for instance, 1 × 3 followed by 3 × 1 convolutions. This is a key feature of the InceptionV3 (18) architecture. As a consequence, the neural net can learn more complex and subtle features, in addition to using fewer parameters. The use of auxiliary classifiers, which are intermediate softmax layers used in the deeper parts of the neural net, is another important aspect to counter vanishing gradients and regularization. Figure 6 below illustrates the InceptionV3 architecture.

**Figure 6:**
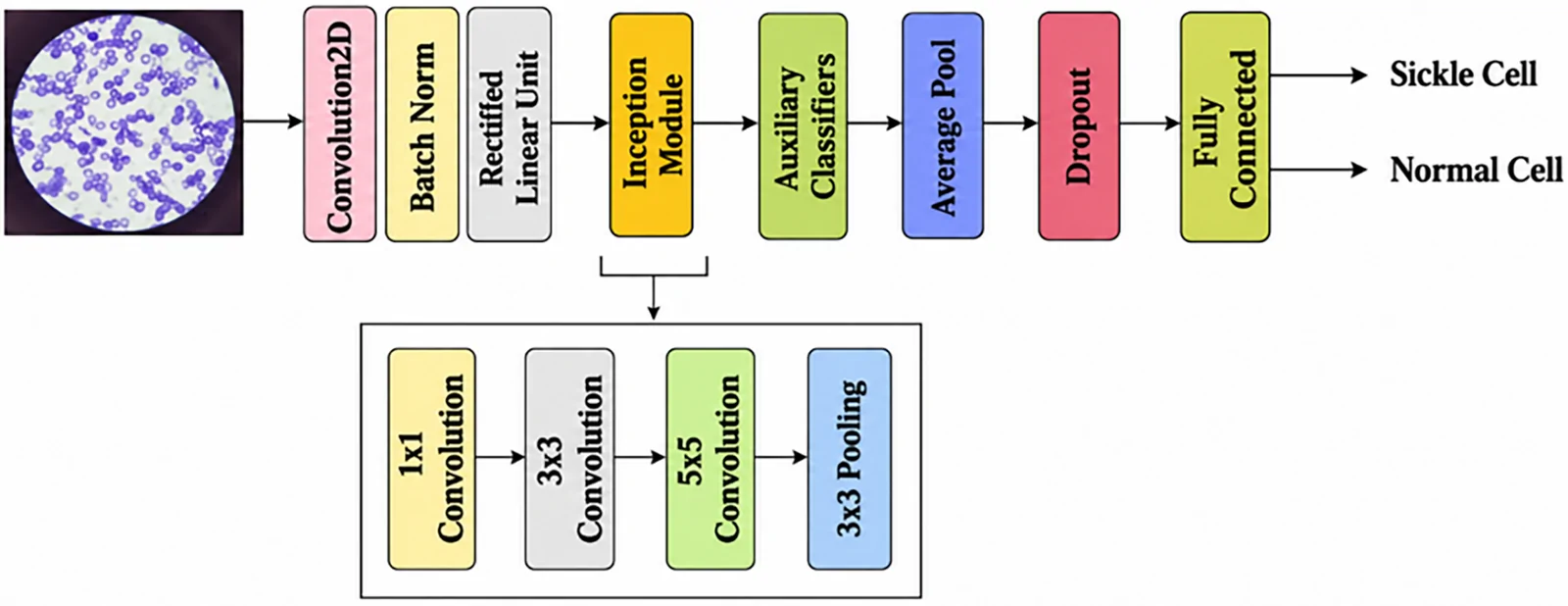
The structure of the InceptionV3 model.

Due to the modularity of the inception block, multiple pooling layers enable multi-scale feature extraction by aggregating the results of convolution layers having different kernel sizes. Thus, the model can simultaneously gather both local and global contextual information, making it highly effective in applications involving features of widely varying scales, including object detection, scene classification, and, in particular, medical imaging. The mathematical form of the Inception Module is mentioned in Equation 7.

$$Y=Concat(W_{1\times 1}^{\left(1\right)}a,\;W_{3\times 1}(W_{1\times 3}(W_{1\times 1}^{\left(2\right)}\;a)),\;W_{3\times 3}^{\left(2\right)}(W_{3\times 3}^{\left(3\right)}(W_{1\times 1}^{\left(3\right)}\;a)),\;W_{1\times 1}^{\left(4\right)}(Pool(a)))$$ (7)

where, $W_{1\times 1}^{\left(1\right)}a$ - 1 × 1 convolution operations to reduce dimensionality, $W_{3\times 1}(W_{1\times 3}(W_{1\times 1}^{\left(2\right)}\;a))$ – Two 3 × 3 factorized convolution operations, $W_{3\times 3}^{\left(2\right)}(W_{3\times 3}^{\left(3\right)}(W_{1\times 1}^{\left(3\right)}\;a))$ - Two 3 × 3 convolution operations for replacing traditional 5 × 5 convolution operations, $W_{1\times 1}^{\left(4\right)}(Pool(a))$ - Pooling operation followed by 1 × 1 convolution operations, Concat(.) - Concatenation of feature maps channel-by-channel from all branches.

The multi-branch design of InceptionV3 makes it an excellent teacher model in Knowledge Distillation (KD) since it captures both local and global variables that are essential for sickle cell image classification. Because of its efficiency, it can guide lightweight student models while maintaining good performance without using excessive resources. It helps students learn subtle differences and improve their generalization skills, which are essential for medical image interpretation, by imparting rich, nuanced knowledge.

##### 3.2.1.4 DenseNet121

DenseNet121 is a deep convolutional neural network that improves information flow and feature reuse across layers. It is based on the idea of Densely Connected Convolutional Networks. It is organized into four thick blocks, each with a varying number of dense layers, and has 121 layers in total (31). The outputs of all previous layers inside a single block are concatenated and sent to each layer in this design (32). A prevalent issue in very deep networks, the vanishing gradient problem is lessened by this dense connectedness, which guarantees maximal information propagation. The architecture of DenseNet allows each layer to directly access the feature maps from all previous layers, greatly enhancing feature reuse and learning efficiency in contrast to typical CNNs, where each layer is simply connected to the next. Mathematically, the output of the l^th^ layer is presented in Equation 8. The structure of the DenseNet121 model is visualized in Figure 7.

$$Y=H(\left[X_{0,}\;X_{1,\;}X_{2,}\ldots \ldots X_{l-1\;}\right])$$ (8)

where, $X_{i}$ – Ouptut of the i^th^ layer, H() - composite function of Batch Normalization → ReLU activation → Convolution operation, $\left[X_{0,}\;X_{1,\;}X_{2,}\ldots \ldots X_{l-1\;}\right]$ - concatenation of feature maps generated from the previous layers.

**Figure 7:**
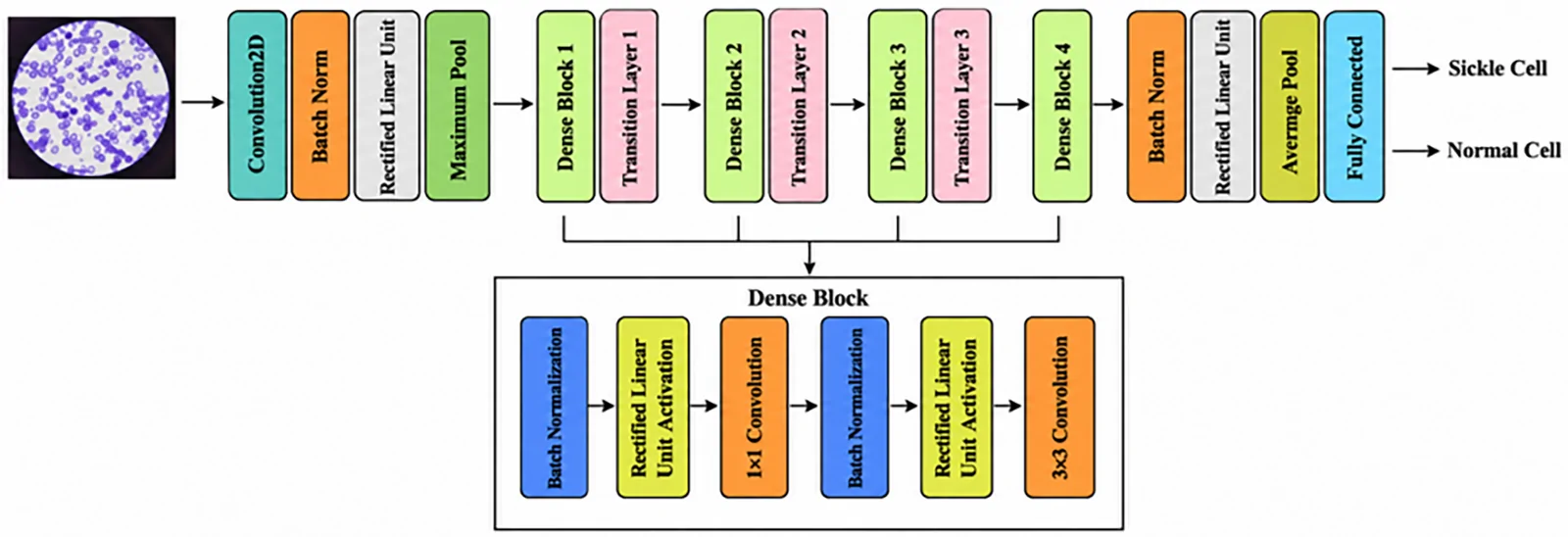
The structure of the Densenet121 model.

DenseNet121 can learn richer, more discriminative features with fewer parameters and smoother gradient flow due to this connectivity pattern. Because of these characteristics, DenseNet121 will be a very good teacher model for the Knowledge Distillation process. It can effectively guide smaller student models in learning specific, clinically relevant features because of its strong generalization and capacity to detect subtle morphological cues in images of sickle cells and healthy blood cells.

#### 3.2.2 Student model

We created unique lightweight student CNN models using the Depthwise Separable Convolutions (DSC) to guarantee effective deployment in clinical environment with constrained computational resources. DSC breaks down a normal convolution into two processes: pointwise convolution, which combines outputs across channels using a 1 × 1 convolution, and depthwise convolution, which applies a single filter per input channel. Compared to conventional convolutional layers, this structural separation significantly lowers the computational complexity and parameter count, allowing for quicker inference and less memory usage (33, 34). The mathematical form for the computational complexity comparison between the standard and Depthwise Separable Convolutions operations is mentioned in Equations 9–13.

$$Cost_{SC}=\;S_{F}\times \;S_{F}\times K\times K\times M\times \;N$$ (9)

$$Cost_{D}=\;S_{F}\times \;S_{F}\times K\times K\times M$$ (10)

$$Cost_{P}=\;S_{F}\times \;S_{F}\times M\times N$$ (11)

$$Cost_{DSC}=\;Cost_{D}+\;Cost_{P}$$

$$\;\;=\;S_{F}^{2}\times K^{2\;}\times M\times N+\;S_{F}^{2}\times M\;\times N\;$$ (12)

$$\frac{Cost_{SC}}{Cost_{DSC}}=\;\frac{1}{N}+\;\frac{1}{K^{2}}$$ (13)

where, S_F_ – Size of the input feature maps, K – Kernel Size, M, N – Input and Output Channnels, Cost_SC_ – Computational cost of Standard Convolution operation, Cost_DSC_ – Computational cost of Depth-wise Seperable Convolution operation.

To ensure that the model remains compact while preserving its representational power, the proposed student model has been designed in a way that emphasizes efficiency and performance. Global Average Pooling, Fully Connected layers for classification, and repeated usage of SeparableConv2D layers along with MaxPooling2D layers in succession to achieve downsampling are all part of the model's design. In order to enhance generalization and the stability of the training process, regularization techniques such as Batch Normalization and Dropout layers have been utilized. The architectural design of the proposed lightweight model is shown in Table 3 and the corresponding architecture diagram is visualized in Figure 8.

**Table 3:** The architectural configuration of the proposed lightweight student model.

L.No	Layer name and type	Output shape	Computational parameters
Layer1	SeparableConv2D	(None, 224, 224, 32)	155
Layer2	MaxPooling2D	(None, 112, 112, 32)	0
Layer3	SeparableConv2D	(None, 112, 112, 64)	2400
Layer4	MaxPooling2D	(None, 56, 56, 64)	0
Layer5	SeparableConv2D	(None, 56, 56, 128)	8896
Layer6	MaxPooling2D	(None, 28, 28, 128)	0
Layer7	SeparableConv2D	(None, 28, 28, 256)	34176
Layer8	MaxPooling2D	(None, 14, 14, 256)	0
Layer9	SeparableConv2D	(None, 14, 14, 256)	68096
Layer10	MaxPooling2D	(None, 7, 7, 256)	0
Layer11	SeparableConv2D	(None, 7, 7, 512)	133888
Layer12	MaxPooling2D	(None, 3, 3, 512)	0
Layer13	SeparableConv2D	(None, 3, 3, 512)	267264
Layer14	MaxPooling2D	(None, 1, 1, 512)	0
Layer15	GlobalAveragePooling2D	(None, 512)	0
Layer16	Dropout	(None, 512)	0
Layer17	Dense	(None, 256)	131328
Layer18	BatchNormalization	(None, 256)	1024
Layer19	Dropout	(None, 256)	0
Layer20	Dense	(None, 2)	514
Total number of computational parameters generated by the proposed lightweight student model			647741

**Figure 8:**
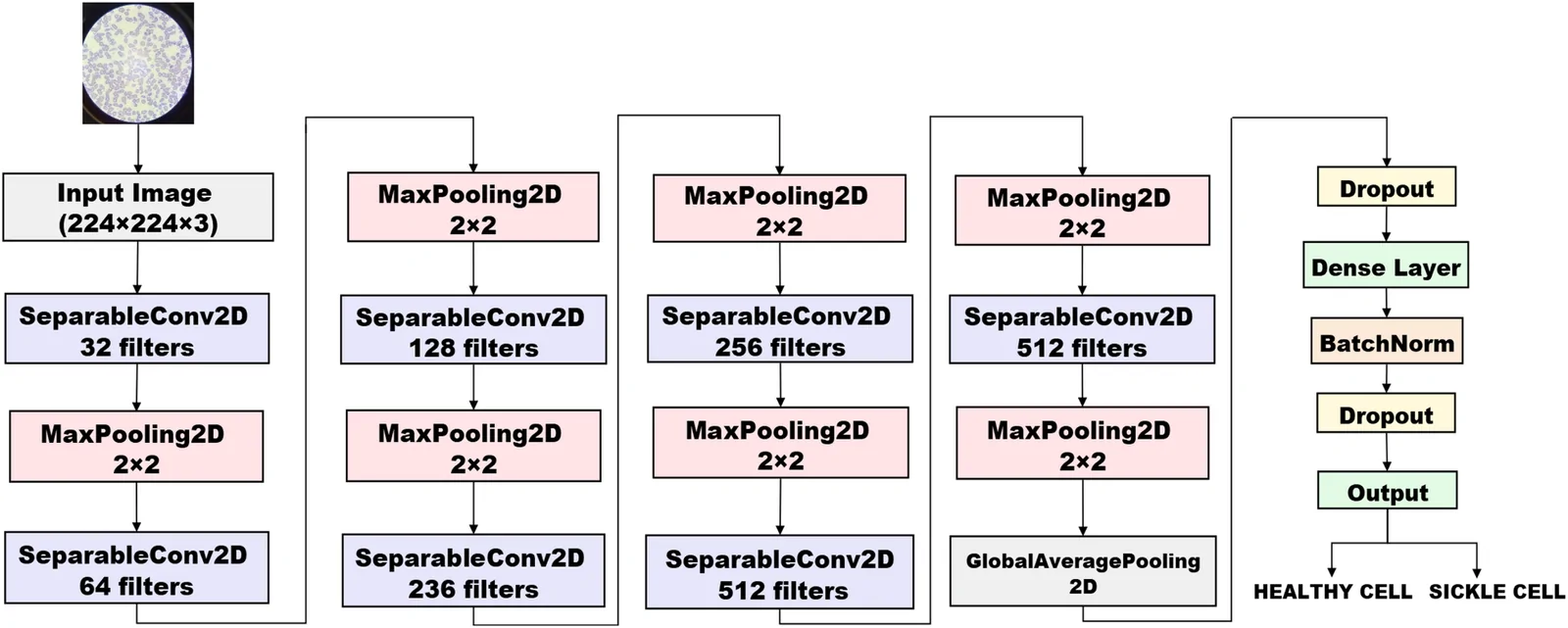
The structure of the proposed lightweight student model.

Table 3 shows that the proposed lightweight model has fewer than 650 K computational parameters, which makes it ideal for deployment in contexts with limited resources. This small computational parameter count represents a substantially lower memory footprint and processing load because the number of computational parameters directly correlates with the size of the model. Its small size guarantees little computing overhead and high classification performance when directed by a more sophisticated teacher model using knowledge distillation. The model is perfect for real-time clinical applications in sickle cell detection because of this balance, which allows for effective learning and high accuracy.

### 3.3 Configuring knowledge distillation

An efficient method for transferring the knowledge acquired by a large, high-capacity teacher model to a small, computationally efficient student model is called Knowledge Distillation (KD). KD makes use of teacher-produced soft labels, such as the class probability distributions, which encode more detailed information about uncertainty and inter-class interactions, rather than relying only on hard ground-truth labels for training the student (35). The student can more successfully generalize and better imitate the teacher's actions due to this dual-supervision system. The Knowledge Distillation process is visualized in Figure 9.

**Figure 9:**
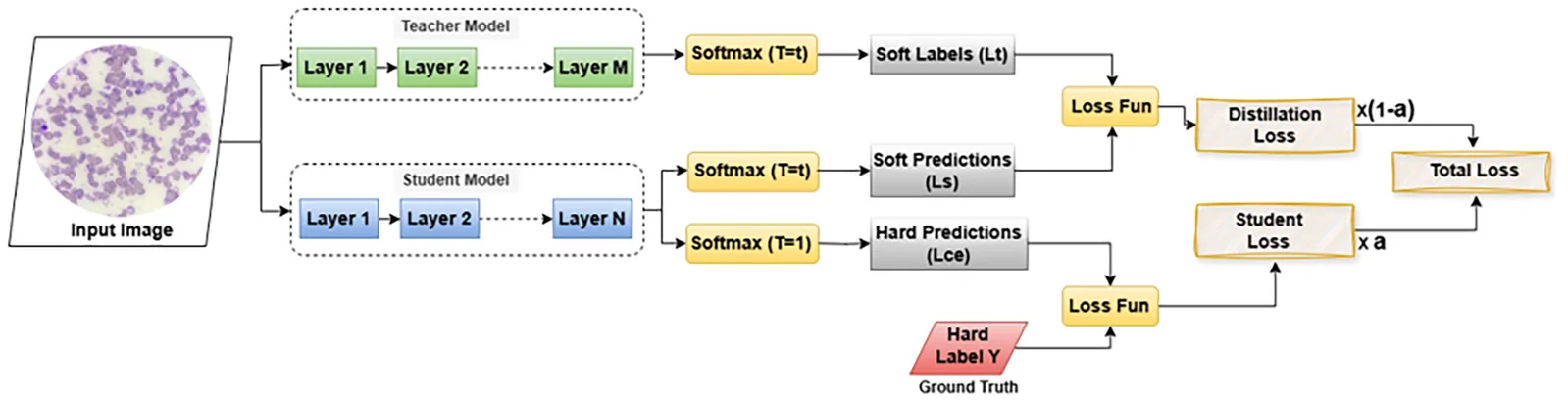
The work-flow diagram to visualize the process of knowledge distillation.

Typically, the distillation process entails optimizing a combined loss function that incorporates the Kullback-Leibler (KL) divergence between the teacher's and student's softened output distributions (soft targets) and the cross-entropy loss between the student's predictions and the true labels (hard targets). The temperature parameter T, which regulates the logits’ softness and is mentioned in Equation 14, modulates this. The output distributions are smoothed by a higher temperature T, which helps students detect subtle class similarities that might not be apparent from one-hot encoded labels alone (36).

$$L_{KD}=a\cdot L_{CE}(gt,\;P)+\;(1-a)\cdot T^{2}\cdot KL\left(\sigma \left(\frac{L_{t}}{T}\right)||\;\sigma \left(\frac{L_{s}}{T}\right)\right)$$ (14)

where, $L_{CE}(y,\;P_{s})$ - Cross-entropy loss between student predictions P and ground-truth labels gt, KL(⋅||⋅) - Kullback-Leibler divergence between the soft outputs of the teacher and the student, $L_{t}$, $L_{s}$ - Logits from the teacher and student networks, respectively, σ(⋅) - Softmax function, T - Temperature hyperparameter, a - Balancing coefficient between the two loss terms.

Algorithm 1 **: Simulating the Knowledge Distillation (KD) Process**

**Input:**

T_model ← Pretrained teacher model

S_model ← Lightweight student model

T ← Temperature paramete*r* = 3

a ← Soft loss weight, where 0 < a < 10

*β* ← Hard loss weight, where *β*=1−a

D ← Training dataset D={(X,Y)} with input images X and ground truth labels Y

N ←: Number of training epochs

**Output:**

Trained student model S_model

**Step 1: Initialize Training Loop**

 1.1. for each epoch e = 1 to N:

 1.2. for each mini-batch (xbatch, ybatch) ∈ D:

**Step 2: Forward Pass**

 2.1. Compute teacher logits:

   logits_teacher ← T_model(x_batch)

 2.2. Compute student logits:

   logits_student ← S_model(x_batch)

**Step 3: Generate Soft Targets using Temperature Scaling**

 3.1. soft_teacher ← Softmax(logits_teacher/T)

 3.2. soft_student ← Softmax(logits_student/T)

**Step 4: Compute Distillation Losses**

 4.1. Soft loss (knowledge loss):

   Lsoft = KLDiv(soft_student,soft_teacher)  ×  T2

 4.2. Hard loss (true label supervision):

   Lhard = CrossEntropy(logits_student,y_batch)

**Step 5: Combine Total Loss**

   5.1. Compute total loss:

      LKD = a⋅Lsoft + *β*⋅Lhard

   5.2. Backpropagate and update S_model weights using LKD

**Step 6: Return Final Model**

 6.1. After EEE epochs, return the trained S_model

Modern pre-trained CNNs like ResNet50, DenseNet121, MobileNetV2, and InceptionV3 are used as teacher models in our study because of their strong feature extraction capabilities. With its lightweight architecture that employs Depthwise Separable Convolutions (DSC), the proposed student network is ideal for low-resource environments like embedded medical devices. KD allows the student to accurately mimic the teacher's performance, resulting in high classification accuracy with a notably lower computing cost. This method strikes a balance between efficiency and performance, which makes it especially suitable for real-time clinical diagnostics, such as the identification of sickle cell disease from microscopy images. The step-by-step procedure for incorporating the Knowledge Distillation process is shown in Algorithm 1.

### 3.4 Building a hybrid Bi-lightweight KD-guided CNN framework-based sickle cell detection system

To enhance the precision and effectiveness of sickle cell detection, the proposed framework presents a hybrid Bi-CNN lightweight model enhanced with Knowledge Distillation (KD) and Machine Learning (ML) approaches. Two lightweight CNN branches are independently trained under the guidance of different high-capacity teacher models to learn complementary representations. In each student model, the Global Average Pooling (GAP) layer (Layer 15) serves as the feature extraction layer and generates a 512-dimensional feature representation. The extracted features from the two distilled student models are subsequently concatenated and provided to an ML classifier for final decision-making. This hybrid design combines complementary distilled feature representations with ML-based classification while maintaining a low computational overhead, supporting potential deployment on resource-constrained edge devices.

#### 3.4.1 Underlying machine models used a hybrid approach

We chose four strong and varied machine learning algorithms, which are XGBoost, Support Vector Machine (SVM), K-Nearest Neighbors (KNN), and Random Forest Algorithms, to enhance the classification abilities of the knowledge-distilled student models. While SVM successfully manages high-dimensional, non-linearly separable spaces using kernel tricks, essential for identifying minute morphological differences in sickle cell images, XGBoost provides robust learning with regularization for deep feature-based tabular data. By combining several decision trees, Random Forest offers robust generalization and noise resistance, whereas KNN offers simplicity through local proximity-based classification (37).

##### 3.4.1.1 XGBoost classifier

A scalable and reliable ensemble learning method based on gradient-boosted decision trees is Extreme Gradient Boosting (XGBoost) (38). Through the progressive addition of trees, each of which corrects the residual errors of its predecessors, it creates a powerful classifier. To minimize overfitting, which is critical in delicate medical applications such as sickle cell disease detection, XGBoost integrates regularization into its objective function.

High-level deep features taken from the Bi-lightweight KD-guided CNN student models are fed to XGBoost in this work. The XGBoost classifier uses these features as input since they encode intricate red blood cell morphological patterns. Modeling the minor differences needed for accurate sickle cell categorization is made possible by its capacity to handle high-dimensional, non-linear feature spaces. Furthermore, its ability to withstand noise enhances its dependability in actual biological imaging applications. The model prediction using the XGBoost algorithm for the input image X is mathematically defined in Equation 15.

$$\hat{Y}=\;\overset{N}{\underset{i=1}{\sum }}\;f_{i}(X_{j}),\;\;\;\;\;f_{i\;}\in F$$ (15)

where $\hat{Y}$ - Predicted output for the X_j_^th^ input sample, X_j_- Extracted feature vector from Bi-lightweight KD guided CNN for the X_j_^th^ input sample, f_i_ – The i^*t*h^ regression tree in the ensemble, F - Functional space of all possible regression trees, N - Total number of boosting rounds (trees).

##### 3.4.1.2 SVM classifier

Support Vector Machine (SVM) is a robust supervised learning algorithm that performs exceptionally well in high-dimensional spaces. Moreover, it works very efficiently in binary and multi-class classifications (39). In the proposed framework, SVM acts as a downstream classifier applied to the deep features extracted from Bi-lightweight KD-guided CNN student models. The deep features encapsulate highly complicated morphological features of sickled versus non-sickled cells.

SVM searches for an optimal hyperplane in the high-dimensional feature space, which separates different classes as much as possible in the feature space in our problem setup of sickled and non-sickled cells (39). Kernel functions of SVM allow the algorithm to recognize non-linear boundaries that generally exist in high-dimensional medical image datasets. This increases the performance of the algorithm significantly (40). Equation 16 defines the SVM decision function's mathematical form.

$$f(X)=\;W^{T}X+b$$ (16)

where f(x) - Decision function used for classification, X - Extracted feature vector from Bi-lightweight KD guided CNN for the X^th^ input sample, W - Weight vector learned by the SVM, b - Bias term.

SVM is efficient in separating overlapping feature distribution due to differences in sizes and morphological features of cells in the problem of sickle cell classification. Its inclusion in the framework increases classification accuracy while retaining computational efficiency.

##### 3.4.1.3 KNN classifier

K-Nearest Neighbor is an example-based, non-parametric algorithm that employs the class label of its closest neighbors in the feature space to predict the label of new data instances (41). It is a simple and efficient downstream classifier. It uses the discriminative deep features extracted from the dual-student models of the proposed Bi-Lightweight KD-guided CNN architecture for sickle cell classification.

For computing the distance between two samples in the feature space, K-NN adopts the Euclidean distance. The most frequent label in the K neighbors is considered. This method is very simple and easy; therefore, it can easily capture the local structure of the data, which is highly beneficial when discriminating sickled red blood cells from normal ones with minor differences. The mathematical expression of K-NN classification is presented in Equation 17.

$$\hat{Y}=mode(\left\{Y_{i}|\;X_{i}\in \;N_{k}(x)\right\})$$ (17)

where $\hat{Y}\;$ - Predicted class label for the input sample *x*, $N_{k}(x)$ - A set of k nearest neighbors of x, $Y_{i}$ - Class labels of the neighbors. KNN is particularly helpful in sickle cell identification because of its adaptability and simplicity of usage, which makes it a perfect option for diagnostic settings with minimal resources.

##### 3.4.1.4 Random forest classifier

A powerful ensemble learning technique called Random Forest (RF) builds several decision trees during training and outputs the mode of each tree's predictions for classification tasks (42). By utilizing the high-level deep features produced by the dual student networks, Random Forest functions as an efficient downstream classifier in the proposed Bi-lightweight KD-guided CNN framework for sickle cell diagnosis.

By choosing feature subsets and data samples for every tree, RF adds randomness, which improves generalization and reduces overfitting. This is particularly useful for feature vectors derived from biological images, which may contain noise or redundant information. It is appropriate for detecting crucial morphological differences in blood cells due to its capacity to manage high-dimensional feature spaces and evaluate feature relevance. Equation 18 defines the mathematical form for the Random Forest's classification result.

$$\hat{Y}=mode\;\{h_{1}(X),h_{2}(X),\ldots \ldots ..h_{n}(X)\}$$ (18)

where $\hat{Y}$ - Predicted class label for input sample X, $h_{i}(X)$ - Output of the i-th decision tree, N - Number of decision trees in the forest

#### 3.4.2 Integrating the Bi-lightweight KD guided models with different teacher models and machine learning classifiers

To combine the complementary representational power of different deep learning models, the proposed Bi-lightweight KD-guided CNN approach creates a two-stream student network by pairing two high-performing teachers from ResNet50, DenseNet121, InceptionV3, and MobileNetV2. In this pair, two lightweight student CNNs independently distilled through two different teacher models acquire various and complementary feature representations. Feature extraction is performed by the Global Average Pooling (GAP) layer (Layer 15 in Table 3), which produces a 512-dimensional representation. Each 512-dimensional feature representation of both student models is concatenated into a unified vector that contains complementary knowledge from both teacher networks. Eventually, this fused feature vector is input into a traditional machine learning classifier [K-Nearest Neighbour (KNN)]. This integrated approach uses the powerful representation of knowledge distillation CNNs along with the machine learning classifiers to achieve an efficient sickle cell detection system.

Algorithm 2 **: Training and Selecting the Best ML Classifiers Using Concatenated Deep Features**

**Input:**

- Student_Model_1, Student_Model_2 ← Top-2 performing distilled student CNN models

- ML_Classifiers = {XGBoost, SVM, KNN, Random Forest}

- Train_Images, Test_Images ← Labeled datasets with image and ground-truth annotations

**Output:**

- Performance metrics for each classifier

- Best-performing ML classifiers based on evaluation metrics

**Step 1: Feature Extraction**

1.1 Load ***Student_Model_1*** and ***Student_Model_2*** in evaluation mode

 1.2 Preprocess all images in Train_Images and Test_Images (resize, normalize)

1.3 Remove the final classification layers from both student models

 1.4 Extract deep features ***F1_train***, ***F2_train*** from Train_Images using the two models

 1.5 Extract deep features ***F1_test***, ***F2_test*** from Test_Images

 1.6 Concatenate corresponding feature vectors:

   - F_train = Concat(F1_train, F2_train)

   - F_test = Concat(F1_test, F2_test)

**Step 2: Prepare Ground-Truth Labels**

2.1 Extract labels ***Y_train***, ***Y_test*** from Train_Images and Test_Images

**Step 3: Train and Evaluate ML Classifiers**

3.1 ***for*** each classifier C ***in*** ML_Classifiers ***do***:

  a. Initialize C with optimized hyperparameters

  b. Train C on (F_train, Y_train)

  c. Predict labels: Y_pred = C.predict(F_test)

  d. Compute evaluation metrics (Accuracy, F1-score)

 e. Store performance metrics for C

**Step 4: Select Best Classifiers**

4.1 Rank classifiers based on primary metric (e.g., F1-score or Accuracy)

 4.2 Select the top-performing classifier ***C_best*** with the highest performance

**Step 5: Return Result**

  -***return (****performance* metrics for all classifiers, C_best)

Through the use of lightweight CNN architectures with effective components like depth-separable convolutions and dual-stream configurations, student models and high-capacity teacher models can be approximated at much lower computational costs. Although student models may perform slightly worse than high-capacity teachers individually, machine learning classifiers effectively mitigate this disparity. This classifier enhances decision-making by utilizing fused feature representations, yielding a classification performance that is more computationally efficient than that of a single high-capacity teacher model. The step-by-step procedure for building and selecting the Best ML Classifiers Using Concatenated Deep Features is mentioned in Algorithm 2, and the step-by-step procedure for predicting the image is shown in Algorithm 3.

Algorithm 3 **: Predicting Sickle Cell Condition Using Bi-KD Guided Student Framework**

**Input:**

- Image_input ← Input image of shape 224 × 224 × 3

- Student_Model_1, Student_Model_2 ← Trained top-2 KD-guided lightweight student CNNs

- ML_Classifier ← Trained best-performing ML classifier (e.g., KNN or SVM)

**Output:**

- Predicted class label: Normal or Diseased

**Step 1: Preprocess Input Image**

1.1 Resize Image_input to 224 × 224 × 3 if not already in that size

1.2 **Step 2: Feature Extraction from Dual Student Models**

2.1 Load Student_Model_1 and Student_Model_2 in inference mode

 2.2 Remove the final classification layer to create the feature extractor

 2.3 Extract deep features:

   Feature_1 ← Student_Model_1(Image_proc)

   Feature_2 ← Student_Model_2(Image_proc)

 2.4 Flatten or pool outputs to get 1D vectors

 2.5 Concatenate extracted features:

   Feature_concat ← Concat(Feature_1, Feature_2)

**Step 3: Predict Class Using ML Classifier**

3.1 Pass concatenated feature vector to ML_Classifier:

  predicted_Label ← ML_Classifier.predict(Feature_concat)

**Step 4: Return Prediction**

4.1 ***if*** predicted_Label == 0 ***do***:

***return*** “Normal Cell"

 4.2 ***else:***

**return** “Diseased Cell”

## 4 Experimental details

### 4.1 Experimental environment used for developing the underlying deep/machine learning models

Every model utilized in this research is based on TensorFlow and the Keras framework. These models are built and assessed on a local system within the Jupyter Notebook environment, which features an NVIDIA GeForce GTX 1650 GPU with CUDA Version 12.6 support, 8 GB RAM, and 117 GB disk space available. This configuration allows for the effective performance of deep learning tasks, such as feature extraction based on CNNs and classification through machine learning. Jupyter Notebook, despite its modest hardware requirements, allows for smooth integration with Python libraries and offers a flexible environment for experimentation and model tuning.

The dataset is divided into Training and Test sets, as indicated in Table 2. Four pretrained CNN models—DenseNet121, MobileNetV2, InceptionV3, and ResNet50—are utilized for feature extraction and trained on the training set for 25 epochs. A lightweight model is proposed in this work, whose architectural configuration is shown in Table 3. The proposed lightweight model is used as a student model in the Knowledge Distillation process and trained along with the above-mentioned Teacher models for 100 epochs. In the course of training, the validation set serves a key role in observing how well the model performs and in guaranteeing generalization. Following training, standard machine learning classifiers from the Scikit-learn (sklearn) framework—K-Nearest Neighbors (KNN), Support Vector Machine (SVM), and Random Forest, as well as XGBoost from the XGBoost library. The extracted CNN features were used as inputs to these classifiers, which were configured using their default settings without additional hyperparameter optimization. The trained classifiers were subsequently evaluated using the test dataset to assess their classification performance.

The task of predicting sickle cell disease is a binary classification problem that uses one-hot encoded labels, so the Binary Cross-Entropy loss function is used for training the CNN model, since it is suitable for binary-class classification. The Adam optimizer with a learning rate of 0.001 is employed to optimize this loss, ensuring adaptive and efficient convergence. To mitigate overfitting and enhance model generalization, a dropout rate of 0.5 is included within the CNN layers. To assess real-time inference latency under memory and compute restrictions, we used the Raspberry Pi 4 Model B device for the edge deployment investigation. This device has a quad-core Cortex-A72 CPU, 4 GB LPDDR4-3200 RAM, USB 3.0 ports, Gigabit Ethernet, and microSD storage. The training parameters and corresponding values used in this research work are mentioned in Table 4.

**Table 4:** The hardware and experimental parameters used to build the underlying models.

S.No	Hardware/experimental parameter	Configuration/value
1	Platform	Jupyter Notebook
2	Disk space	117GB
3	RAM	8GB
4	GPU	NVIDIA GeForce GTX 1650 (4 GB VRAM)
5	CUDA version	12.6
6	Batch Size	32
7	Epochs used local training	100
8	Optimizer	Adam optimizer
9	Learning Rate	0.001
10	Loss function	Categorical_cross entropy
11	Include_top (Backbone Models)	False
12	Trainable (in Transfer Learning)	True
13	Pre-trained Weights	ImageNet
14	Training Epochs for Teacher Models	25
15	Training Epochs for KD process	100
16	KNN Hyperparameters	k = 5, Euclidean distance (Minkowski, *p* = 2), Uniform weights
17	Random Forest Hyperparameters	200 trees, Maximum depth = None, Random seed = 42
18	SVM Hyperparameters	RBF kernel, C = 1.0, Gamma = scale, Probability = True, Random seed = 42
19	XGBoost Hyperparameters	100 estimators, Maximum depth = 6, Learning rate = 0.3, Subsample = 1.0, Column sampling = 1.0

### 4.2 Evaluation metrics

The performance of the underlying deep learning models is evaluated using the following metrics,

1. Accuracy (A)
2. False Positive Rate (FPR)
3. False Positive Rate (FNR)
4. F1-score (FS)
5. Loss (L)
6. Mean Inference Time (MIT)

Accuracy (A) can be defined as the ratio of accurately classified samples out of all samples. The problem with accuracy alone is that it might mislead us in case there is an uneven cost of misclassification. However, other indicators such as False Negative Rate (FNR) and False Positive Rate (FPR) are very important when it comes to getting more information about the performance of our models. FPR is the measure of the percentage of truly negative samples that have been wrongly classified as positive and indicates the risk of a false alarm. FNR is the percentage of truly positive samples that have been classified as negative and indicates the risk of the missed detection. The harmonic mean of precision (P) and recall (R) is used to compute the F1 Score, which gives us one figure that takes into account both indicators. This figure is useful in case of unbalanced classes or when we have to consider both types of errors as important. Binary Cross-Entropy Loss (L) is the difference between the actual binary label and the expected probability in the classification task. Mean Inference Time (MIT), which represents end-to-end latency, is the average amount of time from input image reception to preprocessing, KD-guided CNN feature extraction, and classification using ML algorithms. It imposes harsher penalties for inaccurate confident forecasts. The mathematical forms of these metrics are mentioned in Equations 19–25.

$$A=\frac{TP+TN}{TP+FP+TN+FN}$$ (19)

$$FPR=\;\frac{FP}{FP+TN}$$ (20)

$$FNR=\;\frac{FN}{FN+TP}$$ (21)

$$F1=2\frac{\left(P\times R\right)}{\left(P+R\right)}$$ (22)

$$P=\frac{TP}{TP+FP}$$ (23)

$$R=\;\frac{TP}{TP+FN}$$ (24)

$$L=\;-\frac{1}{N}\;\overset{N}{\underset{i=1}{\sum }}\;TL_{i\;\;}\log\;PL_{i}+(1-TL_{i})\log\;(1-PL_{i})$$ (25)

where, TP – True Positive, TN – True Negative, FP – False Positive, FN – False Negative, N – Number of Instances, TL_i_ – True Label, PL_i_ – Predicted Labels.

## 5 Results and analysis

The analysis of results obtained for classifying sickle cells is subdivided into the following sub-sections.

1. Performance comparison of different Teacher models used in this work
2. Performance comparison of lightweight models with and without Knowledge Distillation
3. Performance Comparison of Hybrid CNN–ML Models Using Single and Bi-Lightweight KD-Guided Feature Extractors
4. Computational Complexity Comparison
5. Latency Analysis on Edge Deployment Focus
6. Performance comparisons on the existing state-of-the-art research
7. Statistical Analysis
8. Explainability Analysis
9. Ablation Study

### 5.1 Performance comparison of different teacher models used in this work

In a knowledge distillation framework, the efficacy of student learning is directly impacted by the teacher's model's ability to generalize and accurately depict feature patterns. Through the evaluation of multiple backbone designs, including InceptionV3, MobileNetV2, DenseNet121, and ResNet50, we are able to determine which model offers the most reliable learning assistance. Analyzing each backbone's performance across metrics such as Accuracy, F1-score, FPR, FNR, and loss helps identify the best balance between predictive power and data richness for guiding student models because each backbone has unique representational strengths and architectural features. Selecting the best teacher models to optimize learning effectiveness and increase student performance requires this comparison.

The classification performance comparison of the four CNN models: MobileNetV2, InceptionV3, DenseNet121, and ResNet50 can be found in Table 5. All models are loaded with pretrained ImageNet weights and then optimized on the Sickle Cell dataset for 25 epochs. By utilizing some critical evaluation measures such as accuracy, F1-score, loss, False Positive Rate (FPR), and False Negative Rate (FNR), their performance was evaluated on the training and test sets. In particular, the DenseNet121 model had the highest generalization and robustness, the highest training accuracy (97.56%), and good testing performance (accuracy – 94.21%, F1-score – 93.57%), and was supported by low FPR and FNR. The second-best testing performance had MobileNetV2, which showed the highest accuracy (93.74%) and F1-score (93.07%). A small difference between training and testing performance proves the reliability and computationally inexpensive nature of this model as a teacher model. However, InceptionV3 demonstrated the lowest level of generalization by showing a considerable decrease in F1-score over the testing time. Compared to DenseNet121, ResNet50 showed somewhat bigger error rate but still had decent consistency. In conclusion, these results prove the importance of selecting teacher models that will preserve high generalization, which is the crucial factor for knowledge distillation.

**Table 5:** Comparative analysis of sickle cell dataset using Pre-trained CNN backbone models as teacher models.

Underlying model	Training dataset					Testing dataset				
	A	FS	L	FPR	FNR	A	FS	Loss	FPR	FNR
	(%)	(%)	(%)	(%)	(%)	(%)	(%)	(%)	(%)	(%)
MobileNetV2	96.11	95.95	0.002	4.38	3.75	93.74	93.07	4.74	6.88	6.25
InceptionV3	94.34	93.21	0.017	6.88	5.62	91.33	90.22	8.28	9.38	8.13
Densenet121	97.56	96.94	0.02	2.50	2.50	94.21	93.57	4.69	6.25	5.62
ResNet50	96.34	95.68	0.029	3.75	3.75	92.05	91.45	5.78	8.75	6.87

A, accuracy; FS, F1-score; L, loss; FPR, false positive rate; FNR, false negative rate.

The graphs of epoch-wise accuracy and F1-score during the training process are depicted in Figure 10 to measure the generalization ability and learning characteristics of the teacher models. Such graphs help to compare the speed of convergence, the stability of the learning process, and the consistency of prediction. The accuracy graphs (Figure 10a) show overall prediction strength, where DenseNet121 and MobileNetV2 have consistently high values over the whole training process. The learning graphs show smooth and stable accuracy growth of these models in comparison with other models. Moreover, the F1-score trends (Figure 10b), which describe the balance between the precision and recall of the models, prove the effectiveness of these two teacher models by demonstrating good and stable progress. Thus, on the basis of this graph's analysis, DenseNet121 and MobileNetV2 can be called the most reliable teacher models for students.

**Figure 10:**
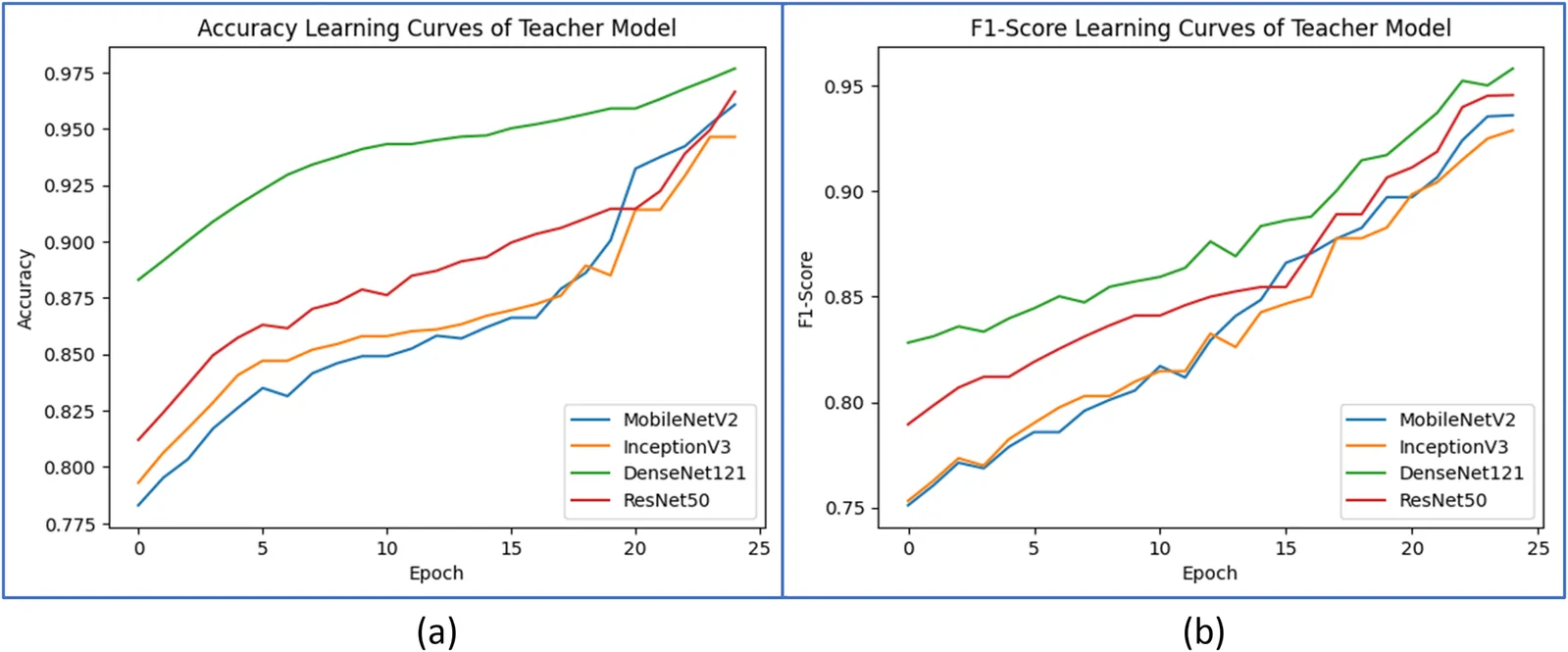
Visualization of accuracy and F1-score learning curves of the different teacher models, **(a)** Accuracy learning curves, **(b)** F1-score learning curves.

### 5.2 Performance comparison of lightweight models with and without knowledge distillation

After the teacher models are built, initialized with pre-trained ImageNet weights, and refined over 25 epochs on the target Sickle Cell Disease image dataset, they provide soft labels in the form of class probability distributions. Small, lightweight student models are trained using these soft labels as valuable targets. A composite knowledge distillation loss function, adjusted by a temperature parameter to soften probability distributions, is used to improve the student model. This loss function incorporates binary cross-entropy (for hard label supervision) and Kullback-Leibler divergence (for soft label alignment). The student can capture both the teacher's encoded sophisticated inter-class knowledge and explicit label correctness using this dual-source learning. To monitor important distillation-specific parameters, including temperature-adjusted validation different metric scores, over 100 training epochs, a customized callback mechanism is configured. Through this paradigm, the resource-efficient student can more easily transfer refined decision boundaries from the high-capacity teacher, improving predicted reliability and model compactness.

In our comparison analysis, we explored the effectiveness of Knowledge Distillation and the effectiveness of different teacher models in guiding the student model. In addition, a baseline student model trained without Knowledge Distillation is also evaluated. Table 6 summarizes these findings and sheds light on how teacher model supervision affects student model performance.

**Table 6:** Comparison of student models’ performance without and with knowledge distillation from different teacher models.

Underlying models with corresponding teacher models	Training dataset					Testing dataset				
	A	FS	L	FPR	FNR	A	FS	Loss	FPR	FNR
	(%)	(%)	(%)	(%)	(%)	(%)	(%)	(%)	(%)	(%)
Student-with-Teacher_MobileNetV2	88.55	87.20	10.72	13.75	9.38	85.35	83.50	13.22	16.25	13.13
Student-with-Teacher_InceptionV3	85.82	84.10	15.05	15.63	12.50	82.82	81.00	18.25	18.13	15.63
Student-with-Teacher_Densenet121	89.09	88.00	11.25	13.75	8.13	85.89	84.20	12.52	15.63	12.50
Student-with-Teacher_ResNet50	87.45	86.05	16.64	14.38	11.25	84.65	83.00	19.64	16.88	14.38
Student-without-KD	66.11	64.30	33.24	28.75	38.75	63.91	62.10	35.24	31.88	40.63

A, accuracy; FS, F1-score; L, loss; FPR, false positive rate; FNR, false negative rate.

Despite having the same architecture, the student models differ in their capacity to extract features based on the teacher model that is employed for knowledge distillation. Performance in classification varies noticeably as a result of this variation. The feature maps produced by student models trained under the direction of several teacher models—MobileNetV2, InceptionV3, DenseNet121, and ResNet50—are visualized in Figure 11 to demonstrate this. To provide a fair assessment of the representational capabilities of each student model, these feature maps are taken from the same layer and filter. Student models directed by MobileNetV2 and DenseNet121 generate feature maps that are sharper and more distinct than those guided by InceptionV3 and ResNet50, as seen in Figure 11 (a1-c1) and (a3-c3). This suggests that DenseNet121 and MobileNetV2 transfer hierarchical feature representations more successfully, allowing the student models to better capture the small morphological differences necessary for precise sickle cell detection. The measures used for downstream classification are directly impacted by these variations in representation quality. Additionally, when analyzing KD-guided student models with the baseline model trained without KD, significant performance improvements are shown across all assessment metrics.

**Figure 11:**
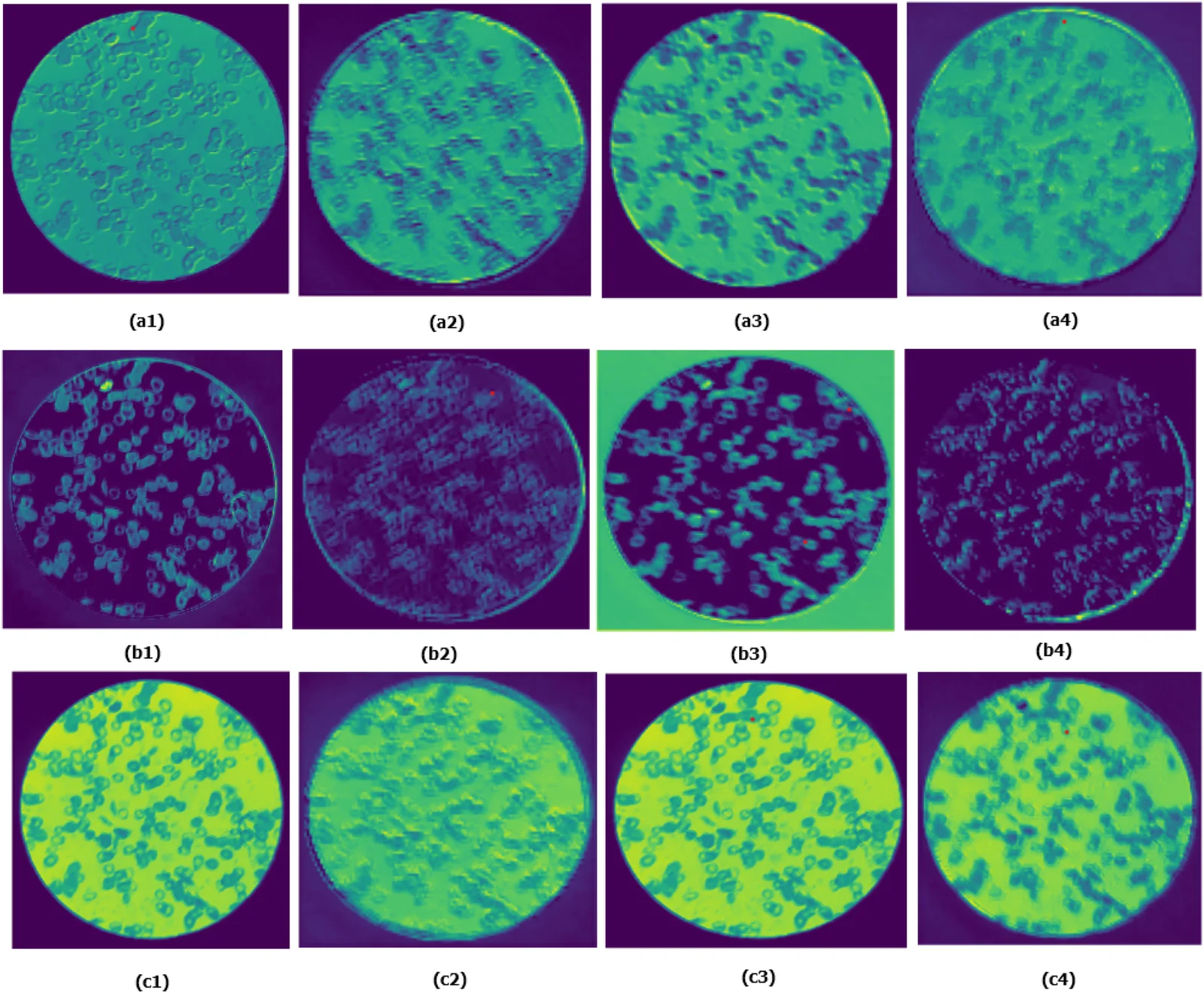
The visualization feature maps generated from the four identical student models guided by the different teacher models (MobileNetV2, InceptionV3, DenseNet121, and ResNet50), (**a1-c1**) – feature maps generated by the student-with-teacher_MobileNetV2, (**a2-c2**) – feature maps generated by the student-with-teacher_InceptionV3, (**a3-c3**) – feature maps generated by the student-with-teacher_DenseNet121, (**a2-c2**) – feature maps generated by the student-with-teacher_ResNet50, (**a1-a4**) (**b1-b4**) (**c1-c4**) - feature maps generated at the same level of layer and filter.

Student models guided by DenseNet121 outperformed student models guided by other teacher models. In terms of Accuracy and F1-score on the testing dataset, the student model KD-guided by the DenseNet121 model increased by 21.98% and 22.1%, respectively, when compared to the student model without KD guidance. Additionally, it produced significant decreases in Loss (22.72%), FPR (16.25%), and FNR (28.13%), highlighting the efficacy of DenseNet121's hierarchical knowledge transmission. Comparably, the MobileNetV2-guided student model demonstrated remarkable improvements, achieving a loss reduction of more than 22%, a 21.4% increase in F1-score, and a 21.44% increase in Accuracy while keeping relatively low FPR and FNR. While ResNet50 and InceptionV3 both improved the performance of their respective student models, they did so in slightly less noticeable ways, particularly in loss reduction. This investigation highlights how the choice of teacher model has a significant impact on the efficacy of knowledge distillation by influencing the caliber of features that students obtain.

The student model trained without KD performed significantly less well on all metrics, exhibiting the greatest error rates (FPR: 31.88%, FNR: 40.63%), the lowest Accuracy (63.91%), and the highest loss (35.24%). These findings demonstrate the efficacy of the KD framework, which greatly improves diagnostic accuracy in the classification of sickle cell disease by allowing even lightweight student models to inherit useful decision boundaries and generalization abilities from high-capacity teacher networks. The learning curves of Accuracy and F1-Score for the student models guided by different teacher models and the student model without KD-process are visualized in Figure 12.

**Figure 12:**
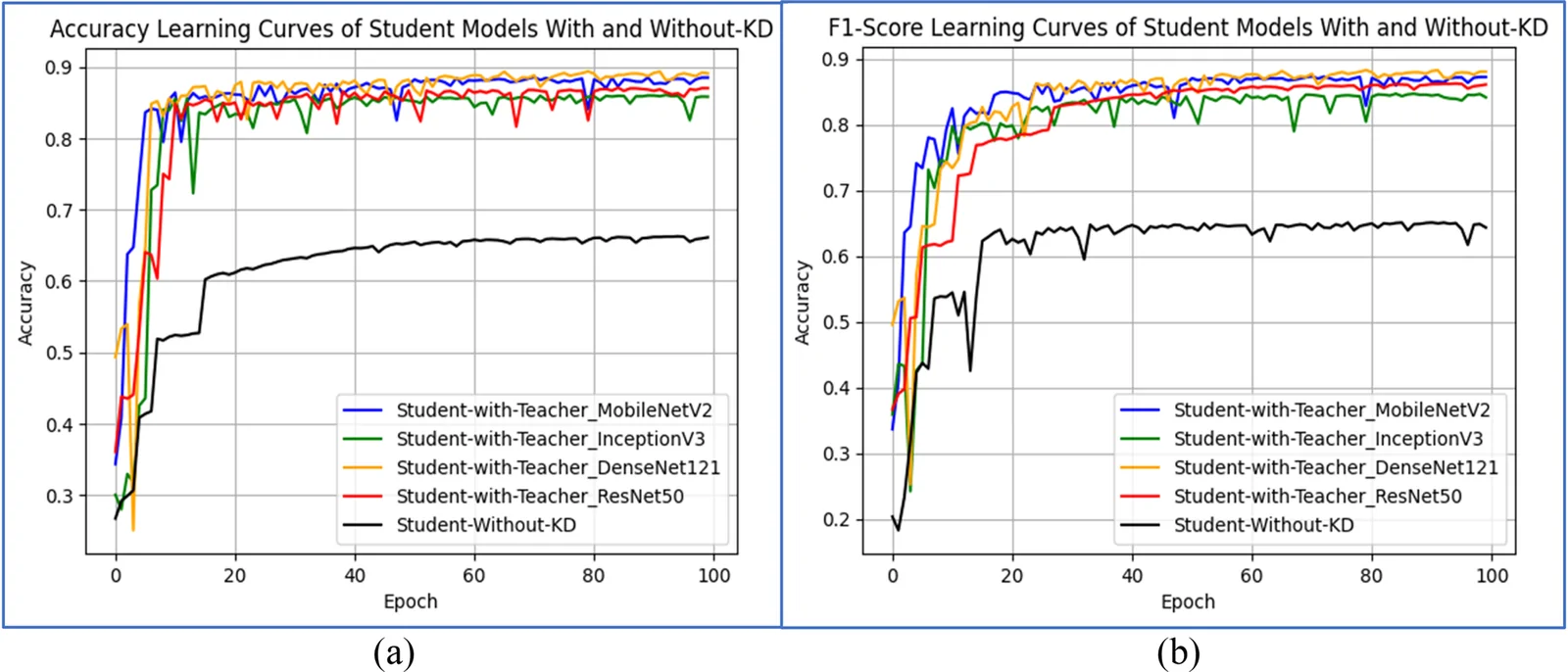
The visualization of accuracy and F1-score learning curves of the student model without KD and with KD from different teacher models, **(a)** Accuracy learning curves, **(b)** F1-score learning curves.

Figures 12a,b show the comparative learning curves of Accuracy and F1-Score, which amply illustrate how well the knowledge distillation (KD) framework helps to improve student models’ Accuracy and F1-score performance. In terms of Accuracy and F1-scores during training, the DenseNet121-derived KD-guided student model consistently performs better than the others, with the MobileNetV2-guided student coming in second. On the other hand, the student model that was not trained with KD performs noticeably less, exhibiting less steady learning behavior and slower convergence. The efficiency and generalization advantages provided by the distillation process are demonstrated by the notable fact that KD-guided models achieve optimal performance significantly earlier and retain a smoother training curve.

The confusion matrices in Figure 13 demonstrate how well the Knowledge Distillation (KD) procedure works to enhance the performance of the student model. To illustrate this, we reviewed confusion matrices of two better-performing KD configurations, Student-with-Teacher_MobileNetV2 and Student-with-Teacher_DenseNet121. Both KD-enabled models (Figures 12b,c) exhibit definite improvements over the standalone student model without KD (Figure 12a): False Positives decrease from 51 to 26/25 and False Negatives decrease from 65 to 21/20, while True Positives rise from 95 to 139/140 and True Negatives from 109 to 134/135. This demonstrates how well KD transfers the teacher's broader feature knowledge, resulting in more balanced and accurate classification. The DenseNet121-guided student model performs slightly better than the MobileNetV2-guided student model, indicating a larger capacity for knowledge transfer.

**Figure 13:**
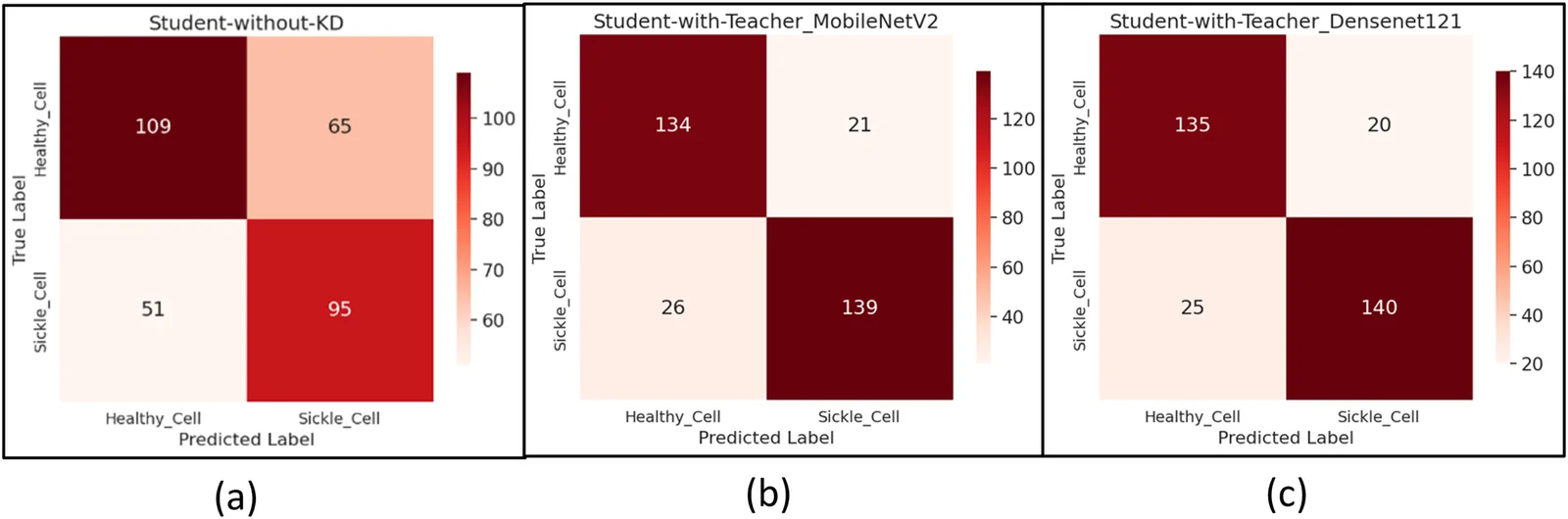
Sample confusion matrix of the representative models, **(a)** Confusion matrix of the standalone student model without KD, **(b)** Confusion matrix of the student model with KD-MobileNetV2, **(c)** Confusion matrix of the student model with KD-DenseNet121.

### 5.3 Performance comparison of hybrid CNN–ML models using single and Bi-lightweight KD-guided feature extractors

Hybrid architectures that combine CNN-based feature extractors with machine learning classifiers, such as SVM, XGBoost, KNN, and Random Forest, are being investigated to analyze the complementary benefits of deep feature representation and traditional decision boundaries. Compared to standalone CNN classifiers, this integration allows for better generalization and interpretability, especially in scenarios involving unbalanced medical imaging. This section examines the performance of hybrid models that combine two different KD-guided student models (bi-lightweight) and those that are built using features from a single lightweight KD-guided student model. Finding out if ensemble-type feature fusion from several distillation sources provides quantifiable performance improvements across important assessment criteria, including Accuracy, F1-Score, Loss, FPR, and FNR.

Initially, the classification performance of hybrid models, which integrate features from a single KD-guided lightweight student model with traditional machine learning classifiers, such as SVM, XGBoost, KNN, and Random Forest, is assessed. To train the classifiers independently, feature vectors are taken from the student model (without a classification head). The hybrid models are then built by integrating the corresponding classifiers with the trained KD-guided student model. The goal is to determine the best student–classifier coupling and investigate the effects of various teacher-guided distillation techniques on the quality of the extracted features. Table 7 provides an overview of the corresponding classification outcomes for the test dataset obtained by integrating different ML classifiers with each KD-guided student model (trained with distinct Teacher Models).

**Table 7:** Comparison of student models’ performance without and with knowledge distillation from different teacher models.

Underlying models with corresponding teacher models	ML classifiers	Testing dataset				
		A	FS	Loss	FPR	FNR
		(%)	(%)	(%)	(%)	(%)
Student-with-Teacher_MobileNetV2	SVM	92.65	92.28	7.22	8.13	6.25
	XG Boost	91.75	91.38	7.42	8.75	7.50
	KNN	92.85	92.55	6.10	8.13	5.63
	Random Forest	92.25	91.90	7.22	8.75	6.25
Student-with-Teacher_InceptionV3	SVM	89.41	88.91	11.85	11.25	9.38
	XG Boost	89.26	88.73	11.60	10.63	10.00
	KNN	89.63	89.10	11.55	10.63	9.38
	Random Forest	89.27	88.73	11.95	10.63	10.00
Student-with-Teacher_Densenet121	SVM	93.46	93.11	5.92	7.50	5.63
	XG Boost	92.89	92.55	5.92	8.13	6.25
	KNN	93.91	93.58	5.32	7.50	5.00
	Random Forest	92.69	92.33	6.32	8.75	6.25
Student-with-Teacher_ResNet50	SVM	90.97	90.50	12.84	9.38	8.13
	XG Boost	90.73	90.25	12.68	9.38	8.75
	KNN	90.80	90.32	12.58	9.38	8.75
	Random Forest	90.69	90.17	12.76	10.00	8.75

Table 7 shows the classification performance of hybrid models that combine conventional ML classifiers with a single KD-guided student model. The performance of these hybrid models is significantly better than that of their counterparts, independent KD-guided student models (according to Tables 6, 7). KNN and SVM are the classifiers that consistently outperform Random Forest and XGBoost (as shown in Table 7). KNN leads by a small margin because it can capture local data patterns well, while SVM is good at separating complex feature boundaries. Both classifiers are in good alignment with the high-dimensional, discriminative features that are taken from KD-guided models.

DenseNet121 and MobileNetV2 stand out as the best teacher models from Tables 5, 6. Compared to its original version, the DenseNet121-guided student combined with KNN obtains 93.91% Accuracy and 93.58% F1-score, which is an improvement of 8.02% and 9.38%, respectively. In the same way, the KNN-guided MobileNetV2-student achieves 92.85% accuracy and 92.55% F1-score, representing increases of 7.5% and 9.05%, respectively. Due to MobileNetV2's effective representation learning and DenseNet121's feature reuse via dense connections, these results demonstrate the better feature extraction capabilities of both networks. Similarly, using KD-guided student models, SVM consistently shows good accuracy and F1-scores, performing slightly lower than KNN. It is a consistent second-best in Hybrid configurations due to its capacity to handle high-dimensional features.

The hybrid approach, in which student models trained by Knowledge Distillation (KD) from MobileNetV2 and DenseNet121 are further improved using classical machine learning classifiers like SVM and KNN, is shown to be effective overall by the confusion matrices in Figure 14. All hybrid models show a significant increase in True Positives (TP) and a decrease in False Negatives (FN) in comparison to their equivalent KD-only counterparts in Figure 13. This enhancement demonstrates how the hybrid technique can use the more compact and discriminative feature representations acquired by KD to fine-tune the classification bounds. The constant increase in TP and decrease in FN across all configurations suggest improved sensitivity and reliability, which are crucial in medical diagnosis tasks like sickle cell identification, even when there are minor changes in True Negatives (TN) and False Positives (FP). All things considered, the hybrid models produce better and more balanced performance by effectively using the advantages of both deep learning and conventional classifiers.

**Figure 14:**
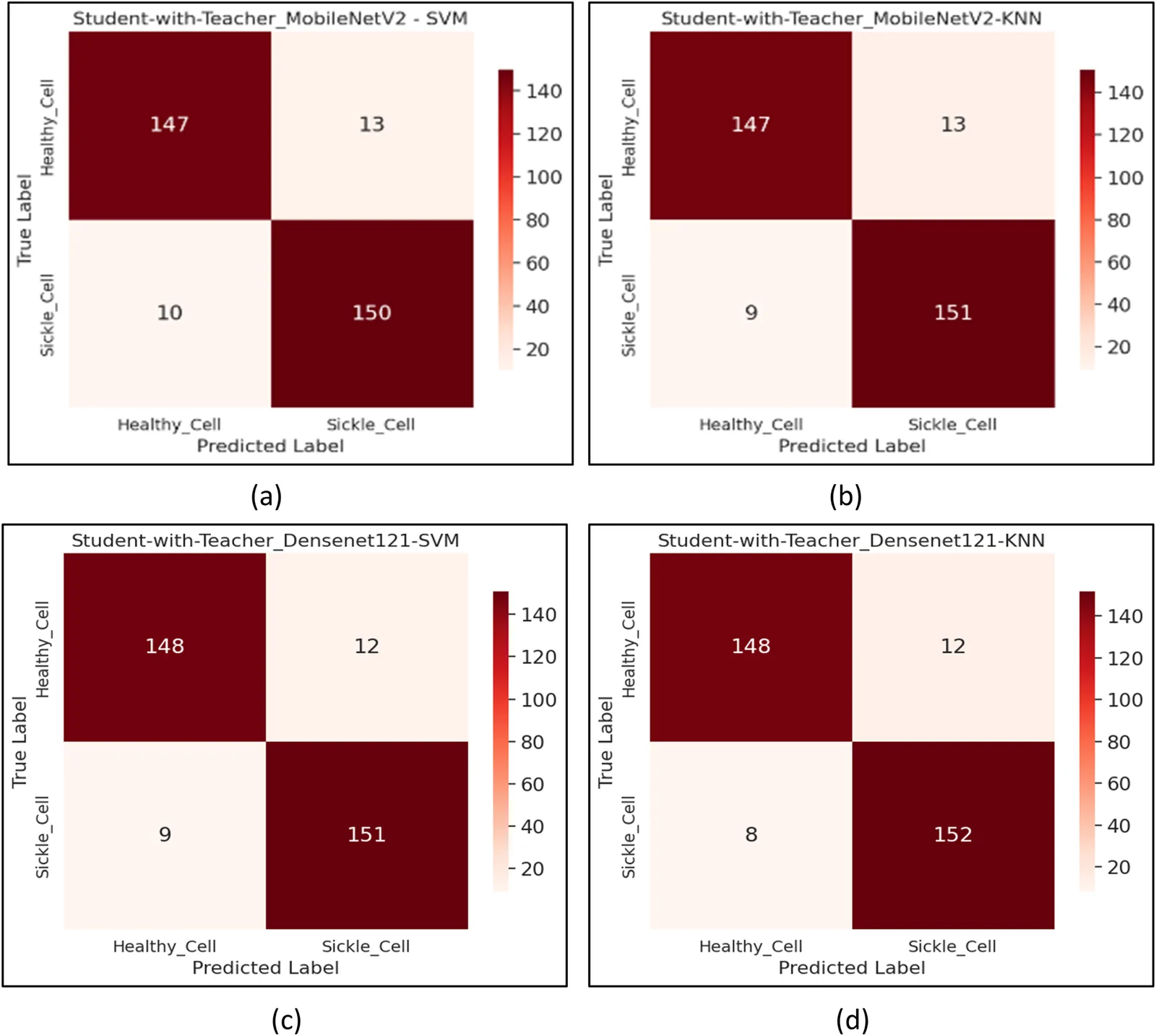
Confusion matrix of the better-performing hybrid models that combine better-performing student models guided by the teacher models MobiLeNetV2 and DenseNet121, **(a)** Student-with-teacher_ MobileNetV2-SVM, **(b)** Student-with-teacher_MobileNetV2-KNN, **(c)** Student-with-teacher_Densenet121-SVM, **(d)** Student-with-Teacher_Densenet121-KNN.

The distinct teacher directing the student model's training shapes its feature extraction even when the architecture of the model stays the same. The student learns a variety of representations because each of the teacher models—ResNet50, DenseNet121, InceptionV3, or MobileNetV2—implements unique inductive biases through distillation. Combining feature maps from two of these student models improves the representation quality because of their different abstractions and complementary capabilities. The generalization capability of the system will be enhanced when better-performing ML classifiers process this augmented feature set. In this context, four KD-guided student models and the top two better-performing ML classifiers (KNN and SVM) are combined in various combinations, as listed in Table 8.

**Table 8:** Performance evaluation of hybrid models combining different Bi-lightweight KD-guided CNN models with SVM and KNN classifiers.

Underlying models with corresponding teacher models	ML classifiers	Testing dataset				
		A	FS	Loss	FPR	FNR
		(%)	(%)	(%)	(%)	(%)
Student-with-Teacher_Densenet121 & Student-with-Teacher_MobileNetV2	SVM	95.08	94.61	3.42	5.62	4.37
	KNN	96.56	96.03	3.37	4.38	3.75
Student-with-Teacher_Densenet121 & Student-with-Teacher_InceptionV3	SVM	93.87	93.33	5.33	7.5	5.1
	KNN	94.34	93.82	4.70	6.88	5.11
Student-with-Teacher_Densenet121 & Student-with-Teacher_ResNet50	SVM	93.83	93.21	4.88	7.5	5.62
	KNN	94.38	93.85	4.23	7.5	4.37
Student-with-Teacher_MobileNetV2 & Student-with-Teacher_InceptionV3	SVM	93.14	92.53	6.53	8.13	5.62
	KNN	93.33	92.71	5.28	7.5	5.62
Student-with-Teacher_MobileNetV2 & Student-with-Teacher_ResNet50	SVM	92.94	92.27	6.24	8.13	6.25
	KNN	93.26	92.61	4.80	8.13	5.62
Student-with-Teacher_InceptionV3 & Student-with-Teacher_ResNet50	SVM	91.28	90.49	13.04	10.01	7.50
	KNN	91.03	90.22	12.79	10.10	8.13
Student-with-Teacher_Densenet121 & Student-with-Teacher_MobileNetV2 & Student-with-Teacher_InceptionV3 & Student-with-Teacher_ResNet50	SVM	95.27	94.73	3.25	5.62	4.37
	KNN	96.85	96.19	3.27	3.13	4.68

All bi-lightweight student model hybrid approaches perform better than their single-student counterparts in terms of accuracy, F1-score, loss, FPR, and FNR, according to a comparison of Tables 7, 8. This is due to two different KD-guided student models teaching complementary and varied aspects that improve generalization and resilience. Loss reductions of up to 50% and Accuracy gains of 1% to 3% are possible. For instance, the DenseNet121 student using KNN gets 93.91% Accuracy and 5.32% loss (Table 7). When combined with MobileNetV2 (Table 7), this improves to 96.56% Accuracy and 3.37% loss, demonstrating a 2.65% Accuracy improvement and a 1.95% reduction in loss. The bi-model approach's efficacy is confirmed by similar trends shown for the F1-score, FPR, and FNR. Further, these improvements occur in Bi-lightweight student model combinations.

The best-performing configuration is the blend of Student-with-Teacher_DenseNet121 and MobileNetV2, especially when combined with KNN, which achieves 96.56% Accuracy and 96.03% F1-score. MobileNetV2's effective, lightweight representation learning and DenseNet121's dense connectivity, which encourages efficient feature reuse, are responsible for this higher performance. Together, they create a highly discriminative feature space that is perfect for KNN-based classification. This bi-lightweight hybrid model produces remarkable results, including 95.08% accuracy and 94.61% F1-score, even when employed with SVM. Its performance roughly matches that of the four-student hybrid model, which has 95.27% accuracy and 94.73% F1-score. The success of this two-model coupling is highlighted by the marginal differences across all important metrics, including accuracy (0.19%–0.29%), F1-score (0.09%–0.18%), loss (0.07%), FPR (0.12%–0.16%), and FNR (0%–0.9%). According to these results, a well-matched bi-lightweight hybrid can provide performance that is almost equal to complex multi-model ensembles, providing a more effective solution with a substantially lower computational cost.

The efficacy of employing four KD-guided CNN models and bi-lightweight KD-guided CNN models in hybrid classification setups is illustrated by the confusion matrices in Figure 15. The Bi-lightweight KD models (Figures 15a,b) consistently outperform the single KD-guided hybrid models in Figure 14—True Positives rise to 154 and False Negatives fall to 6 as opposed to a TP range of 147–148 and FN of 12–13 in Figure 14. This suggests that combining information from two lightweight teacher models produces richer and more varied feature representations, improving the accuracy of sickle cell detection by the downstream SVM and KNN classifiers.

**Figure 15:**
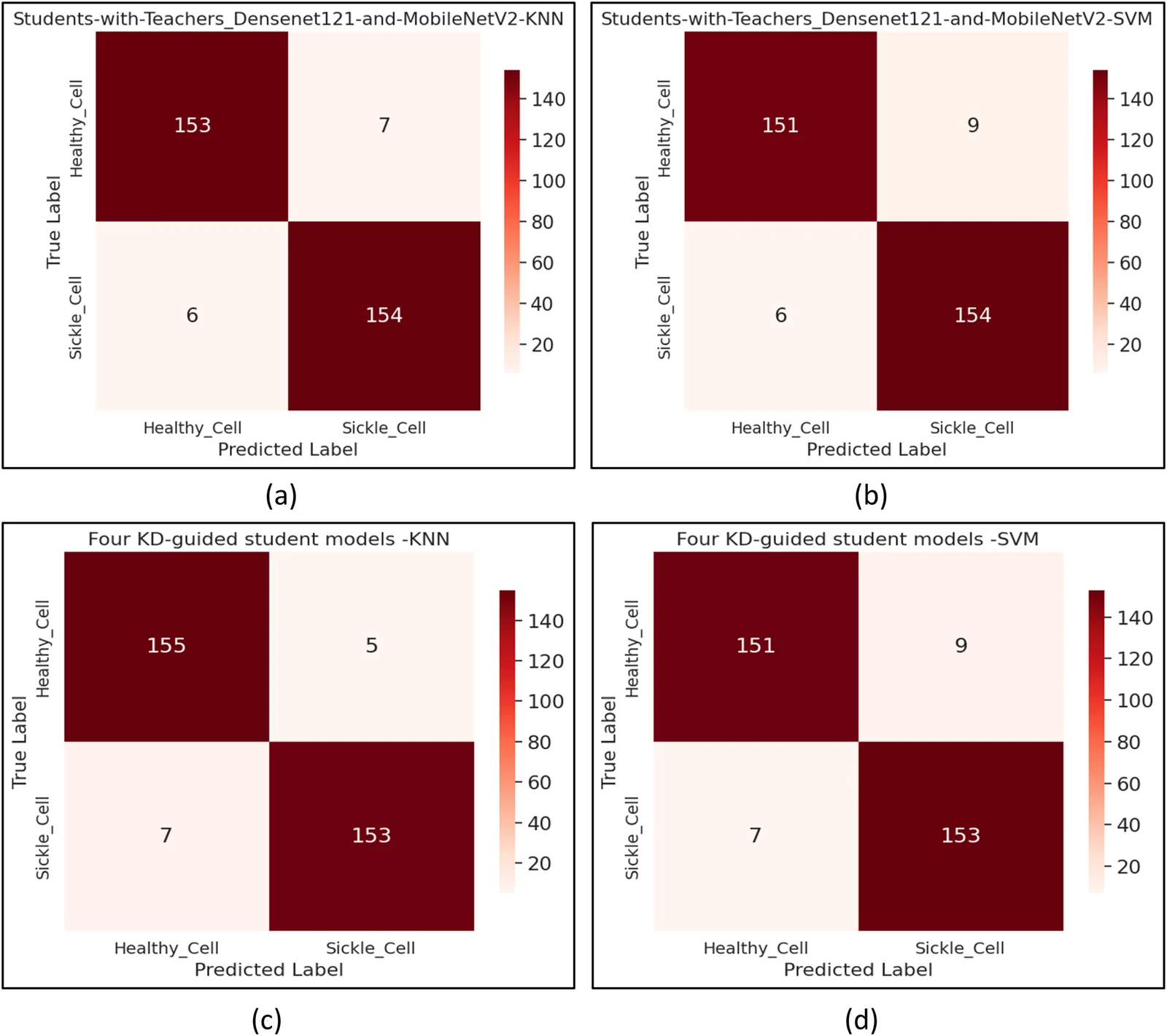
Confusion Matrix of the Hybrid approach with Bi-lightweight KD-guided CNN models and Four KD-guided CNN models with SVM and KNN Classifiers, **(a)** Bi-lightweight KD-guided CNN models with SVM Classifiers, **(b)** Bi-lightweight KD-guided CNN models with KNN Classifiers, **(c)** Four KD-guided CNN models with SVM Classifiers, **(d)** Four KD-guided CNN models with KNN Classifiers.

Additionally, there is no significant difference in the classification performance between the Bi-lightweight KD models (Figures 15a,b) and the Four KD-guided CNN models (Figures 15c,d). The Bi-lightweight models produce slightly better True Positive and False Negative rates (TP: 154 vs. 153, FN: 6 vs. 7), while the Four KD-guided models show slightly higher F1-Score and Accuracy (Table 8). This implies that the Bi-lightweight technique achieves similar outcomes with less complexity while maintaining higher sensitivity—a crucial component in medical diagnostics—even though the four-model ensemble would offer a tiny advantage in aggregate performance metrics. Performance and efficiency are thus strongly traded off in bi-lightweight KD-guided hybrids.

A hybrid student model of Student-with-Teacher_DenseNet121 and Student-with-Teacher_MobileNetV2 with KNN gives 96.56% Accuracy and 96.03% F1-score, thus proving the utility of utilizing complementary knowledge of the two independently distilled students. The improvement compared to teacher models, DenseNet121 and MobileNetV2, which give 94.21% and 93.74% accuracy and 93.57% and 93.07% F1-score, respectively, is up to 2.35% for accuracy and 2.96 percentage points for F1-score. It gives better results in terms of FPR (4.38%) and FNR (3.75%), along with a 3.37% loss. As can be seen in Table 8, the four-student KNN setup gives slightly higher accuracy (96.85%) and F1-score (96.19%), yet the difference between the latter and the former configuration is only 0.29 and 0.16 percentage points, respectively, at the cost of double the number of distilled student branches. Thus, the DenseNet121–MobileNetV2 combination gives the best performance-complexity trade-off and is selected as the Bi-lightweight configuration. A comparative study against multi-teacher configurations utilizing a single student with teacher-loss signal averaging or ensemble is not done in this paper and should be considered in future work.

### 5.4 Computational complexity analysis

The same student model architecture is implemented using conventional convolution techniques to perform a comparative analysis and assess the effect of depth-wise separable convolution on computational complexity. Except for the type of convolution operation used, all configurations are maintained the same (as shown in Tables 3, 4), including the number of layers, parameters, and training setup. Table 9 displays the results of measuring the performance and computational complexity measures, which include accuracy, F1-Score, model size, and computational parameters. Additionally, the metrics achieved by the student model using standard convolution techniques are also compared with two of the best (KD-guided student models, Student-with-Teacher_DenseNet121 and Student-with-Teacher_MobileNetV2) to give a more comprehensive view of the role of the KD process in enhancing the performance of lightweight and less performing student models.

**Table 9:** Comparison of depth-wise separable convolution and standard convolution in student models with and without knowledge distillation.

Model	Testing time performance metrics		Computational complexity metrics			
	A	FS	Model size	NCP	MIT	FLOP
	(%)	(%)	(MB)	(Million)	MS	GFLOP
Student Model-DSC (without KD)	63.91	62.10	6.47	0.65	160	0.237
Stud-Standard Convolution	85.94	85.36	61.24	16.05	140	2.41
Student-with-Teacher_Densenet121 (with DSC)	85.89	84.20	6.47	0.65	100	0.237
Student-with-Teacher_MobileNetV2 (With DSC)	85.35	83.50	6.47	0.65	160	0.237

A, accuracy; FS, F1-score; NCP, number of parameters generated by the model; MIT, mean inference time; GFLOP, giga floating point operations.

Table 9 illustrates the trade-off between computational efficiency and predictive effectiveness by contrasting student models utilizing depthwise separable and ordinary convolutions. The findings in Table 9 demonstrate that the student model using standard convolution outperforms its lightweight counterpart using DSC in terms of accuracy (85.94%) and F1-score (85.36%), but at a high cost, with a model size of 61.24 MB and 16.05 million computational parameters. Furthermore, depthwise separable convolutions (DSC) moderately improve inference time from 160 ms to 140 ms and drastically lower computational load, reducing FLOPs from 2.41 to 0.237 GFLOP (∼90%).

While the accuracy (63.91%) and F1-score (62.10%) are noticeably lower, the student model with DSC significantly decreases the model size to 6.47 MB and computational parameters to 0.65 million. Two high-performing teacher models, DenseNet121 and MobileNetV2, are used to perform knowledge distillation (KD) to solve this degradation. The distilled student models achieve ∼85% accuracy and ∼84% F1-score while retaining the DSC's lightweight characteristics (6.47 MB, 0.65M parameters). This illustrates how well DSC for computational efficiency and KD for performance recovery work together, making these models ideal for use in environments with limited resources.

A hybrid technique that combines Bi-lightweight KD-guided CNN architectures with KNN and SVM classifiers substantially improved the performance of the KD-guided student model beyond that of the individual teacher models. As shown by the examination of FLOPs and mean inference time in Table 10, this combination not only improved classification performance but also reduced the computing complexity.

**Table 10:** Computational complexity comparison among the proposed hybrid approach, individual KD-guided student models, and their corresponding teacher models.

Model	Testing time performance metrics		Computational complexity metrics		
	A	FS	MIT	FLOP	Model size
	(%)	(%)	(ms)	(GFLOP)	(MB)
Student-with-Teacher_Densenet121 (with DSC)	85.89	84.20	100	0.237	6.47
Student-with-Teacher_MobileNetV2 (With DSC)	85.35	83.50	100	0.237	6.47
Densenet121 (Teacher Model)	94.21	93.57	780	2.88	83.41
MobileNetV2 (Teacher Model)	93.74	93.07	400	0.300	26.85
Hybrid Approach with Bi-lightweight KD-guided CNN Models (Guided by Densenet121 and MobileNetV2) with KNN Classifier	96.56	96.03	455	0.343	12.95
Hybrid Approach with Bi-lightweight KD-guided CNN Models (Guided by Densenet121 and MobileNetV2) with SVM Classifier	95.08	94.61	418	0.342	12.95

Although the hybrid technique uses bi-lightweight KD-guided CNN models from two different teacher networks, it maintains an exceptionally low computational footprint, with a FLOP range of only 0.342–0.343 GFLOPs (Table 10). As indicated in Table 10, there is a significant decrease of about 88% when compared to the DenseNet121 teacher model (2.88 GFLOPs), and it is still computationally lighter than MobileNetV2 (0.300 GFLOPs). The hybrid models provide significant savings in model size, occupying only 12.95 MB, which is considerably smaller than DenseNet121 (83.41 MB) and MobileNetV2 (26.85 MB), in addition to reduced FLOPs. This size comes from the combined footprint of the two KD-guided student models (2 × 6.47 MB) and the saved machine learning classifier (about 10 kB). The compact architecture is particularly beneficial for use in memory-constrained edge environments, as it allows for quicker model loading, lower I/O latency, and easy integration into portable diagnostic platforms.

Even in the absence of GPU acceleration, the hybrid model's inference time (420–455 ms) is respectable, significantly faster than DenseNet121 (780 ms), and on par with MobileNetV2 (400 ms). Crucially, this efficiency does not equate to a loss of accuracy; rather, Table 10**:** Computational Complexity Comparison among the proposed Hybrid approach, Individual KD-guided student models, and their corresponding Teacher Models it provides a performance boost, with testing accuracy up to 96.56%, outperforming both individual student and teacher models. Significantly, performance is not affected by this decrease in memory and processing demands; the proposed hybrid approach outperforms the individual student and teacher models in terms of performance improvements. This demonstrates how well the hybrid fusion strategy, which uses classifiers like KNN and SVM, produces diagnostic solutions that are both clinically dependable and resource-efficient.

The KD-guided student models utilized in the proposed hybrid approach were translated to TensorFlow Lite (TFLite) format to facilitate deployment on edge and resource-constrained devices (originally in.h5 to.tflite). Especially when using quantization techniques like Float32, this conversion greatly minimizes the size of the model and speeds up inference. With little effect on the degradation of classification performance, TFLite models are designed for low-latency, low-power operation on devices with constrained computational resources. Because of these benefits, TFLite is a workable solution for clinical real-time applications.

The effects on model size, inference time, and performance of converting the proposed hybrid models from.h5 to TensorFlow Lite (TFLite) Float32 format are shown in Table 11. TFLite conversion achieved a nearly 69% reduction in model size for the KNN-based hybrid model, bringing it down from 12.95 MB to 3.96 MB. With only a 1.11% decline in accuracy (from 96.56% to 95.45%) and a 1.01% drop in F1-score (from 96.03% to 95.02%), robust performance is maintained even though the inference time was cut in half, from 455 ms to 113 ms. A similar pattern is seen in the SVM-based hybrid model, which had its inference time dropped in half from 418 ms to 108 ms, and its model size decreased from 12.95 MB to 3.96 MB. The F1 score fell 1.33% (from 94.61% to 93.28%), and the classification accuracy slightly declined by 1.4% (from 95.08% to 93.68%). According to the experimental results shown in Table 11, the feasibility of using these hybrid models in real-time clinical scenarios is significantly improved by the decrease in model size and response time, especially on devices with limited memory and power.

**Table 11:** Computational complexity comparison of the proposed hybrid approach with its.h5 and. tflite format.

Model	Model format	Testing time performance metrics		Computational complexity metrics		
		A	FS	MIT	FLOP	Model size
		(%)	(%)	(ms)	(GFLOP)	(MB)
Student-with-Teacher_Densenet121 (with DSC)	.h5	85.89	84.20	100	0.237	6.47
Student-with-Teacher_Densenet121 (with DSC)	.tflite	83.55	82.91	60	0.237	1.98
Student-with-Teacher_MobileNetV2 (With DSC)	.h5	85.35	83.50	100	0.237	6.47
Student-with-Teacher_MobileNetV2 (With DSC)	.tflite	83.11	82.45	60	0.237	1.98
Hybrid Approach with Bi-lightweight KD-guided CNN Models (Guided by Densenet121 and MobileNetV2) with KNN Classifier	.h5	96.56	96.03	455	0.343	12.95
Hybrid Approach with Bi-lightweight KD-guided CNN Models (Guided by Densenet121 and MobileNetV2) with KNN Classifier	.tflite	95.45	95.02	113	0.343	3.96
Hybrid Approach with Bi-lightweight KD-guided CNN Models (Guided by Densenet121 and MobileNetV2) with SVM Classifier	.h5	95.08	94.61	418	0.342	12.95
Hybrid Approach with Bi-lightweight KD-guided CNN Models (Guided by Densenet121 and MobileNetV2) with SVM Classifier	.tflite	93.68	93.28	108	0.342	3.96

### 5.5 Latency analysis on edge deployment focus

To deploy CNN-based models on edge devices, where prompt response is crucial for real-time clinical applications, latency analysis is necessary. Superior precision is not enough on its own; low inference latency and deployment feasibility are essential to guarantee that the model works within the power, memory, and computing constraints of the device. Additionally, it assists in locating bottlenecks and refining models to achieve a fair balance between efficiency and performance.

The top-performing Hybrid Approach with Bi-lightweight KD-guided CNN Models (Guided by MobileNetV2 and Densenet121) and KNN Classifier is chosen for this analysis. Both the.h5 and.tflite formats of the KD-guided CNN models are examined for latency to assess their suitability for deployment on devices with limited resources, like a Raspberry Pi. Table 12 provides a summary of the related latency measurements and deployment feasibility findings.

**Table 12:** Comparison of computational complexity analysis in CPU vs. Raspberry Pi Environment.

Model	Model format	MIT		
		CPU	Raspberry Pi	Model size
		(ms)	(ms)	(MB)
Hybrid Approach with Bi-lightweight KD-guided CNN Models (Guided by Densenet121 and MobileNetV2) with KNN Classifier	.h5	206	455	12.95
Hybrid Approach with Bi-lightweight KD-guided CNN Models (Guided by Densenet121 and MobileNetV2) with KNN Classifier	.tflite	113	206	3.96

For both model formats (.h5 and.tflite), Table 12 demonstrates that the Raspberry Pi has a larger inference latency than the CPU. The. tflite model lowers this to 206 ms on Raspberry Pi and 113 ms on CPU, whereas the.h5 model takes 455 ms on Raspberry Pi versus 206 ms on CPU. This illustrates embedded systems’ constrained processing power. However, compared to.h5, the.tflite format almost halved the inference time on Raspberry Pi, allowing for real-time inference. This experiment demonstrates that, despite limited resources, the proposed hybrid model can be implemented on edge devices such as Raspberry Pi, where portability and quick responses are crucial in healthcare applications.

### 5.6 Performance comparisons on the existing state-of-the-art research

The effectiveness of the proposed hybrid model is further supported by comparing its performance with recent state-of-the-art approaches for sickle cell detection. Table 13 presents a comparison between the proposed Hybrid approach and representative state-of-the-art techniques for classifying sickle cell disease according to Accuracy, Loss, F1-Score, FPR, FNR, model size, and computational parameters. The comparison involves traditional deep-learning techniques and DenseNet121-based techniques with various image-enhancement approaches, such as Intuitionistic Fuzzy Index (IFI), Single-Scale Retinex (SSR), and IFDR, in which IFDR is a combination of IFI and SSR. Notably, the studies (43, 44) use the same Sickle Cell Disease Dataset (22) as in the present study and thus provide a more meaningful comparison. The proposed Hybrid approach obtains 96.56% Accuracy and 96.03% F1-Score, marginally outperforming the DenseNet121 + IFDR technique with 96.49% Accuracy and 95.37% F1-Score (43, 44). It is also shown in the IFDR-based study that the combination of IFI and SSR image enhancement with DenseNet121 results in better classification accuracy than the individual image-enhancement component.

**Table 13:** Performance comparison with the existing state-of-the-art works.

S.No	Model	Accuracy	Loss	F1-score	FPR	FNR	Model size	Computational parameters
		(%)	(%)	(%)	(%)	(%)	(MB)	(Million)
1	MaxViT-Tiny (43)	94.2	-	93.7	-	-	-	∼31
2	InceptionV3 (43)	93.0	-	92.4	-	-	-	∼24
3	DenseNet121 + IFI Enhancement Component (44)	90.35	-	86.78	-	-	-	-
4	DenseNet121 + SSR Enhancement Component (44)	93.85	-	91.73	-	-	-	-
5	DenseNet121 + IFDR Enhancement Component (44)	96.49	-	95.37	-	-	-	-
6	Proposed Hybrid Approach	96.56	3.37	96.03	4.38	3.75	12.95	0.343

‘-’: Not addressed in the work.

The proposed method also has significant superiority with respect to model size and computational complexity, with a small model size of 12.95 MB and 0.343 million computational parameters. On the other hand, MaxViT-Tiny and InceptionV3 have approximately 31 million and 24 million parameters, respectively (43). The IFI-, SSR-, and IFDR-based methods do not specify the model size and computational parameters (44). However, these methods use the unmodified DenseNet121 architecture along with additional image-enhancement methods. Thus, it can be assumed that their computational requirements are higher than those of the proposed lightweight framework, although it is impossible to estimate their exact value based on the provided results. The proposed approach achieves such compactness because of lightweight student CNNs enhanced with knowledge distillation and followed by feature fusion and ML classification.

Also, the proposed approach obtains 4.38% FPR and 3.75% FNR, which indicates balanced classification performance, and the 3.37% loss also further confirms the effectiveness of the proposed learning framework. However, the comparison is done using the same public dataset but with different data preprocessing, data partitioning, data augmentation, training process, and evaluation protocol. Hence, the comparison is done in order to contextualize the proposed approach in comparison to previously reported results and not for establishing superiority of the proposed approach.

### 5.7 Statistical analysis

Statistical analysis is essential for confirming the efficacy and clinical dependability of deep learning models in medical image-based diagnosis for sickle cell identification. Common issues like class imbalance and imaging quality fluctuation make it difficult to evaluate whether differences in performance indicators, such as F1-Score, FPR, and FNR, are statistically significant across models. To address these issues, techniques like the *p*-value test are conducted in this study. Confidence intervals, such as the 95% CI, provide information about the accuracy and consistency of important measures. The reliability and diagnostic utility of automated sickle cell detection methods depend heavily on these statistical analyses. Without relying on Accuracy or Loss, a model's efficacy is commonly established if it performs statistically better on important metrics that measure precision, recall, and diagnostic error, such as F1-Score, FPR, and FNR. Our statistical analysis is therefore concentrated on F1-Score, FPR, and FNR since they provide a more clinically significant evaluation of model performance.

We created an augmented test dataset of 750 instances, evenly distributing healthy and diseased cell images, using non-deformation-based augmentation approaches rather than conducting statistical analysis directly on the existing test dataset. These include precise modifications to brightness, saturation, sharpness, contrast, and rotation, as well as transposition, all of which maintain the cells’ underlying morphological characteristics. To support a thorough and objective statistical analysis, the expanded dataset was then evenly divided into five subsets, each containing 150 instances with a balanced distribution of Healthy and diseased samples. Due to the limited number of original test images, the augmented samples were treated as repeated evaluation sets rather than as independent samples. As a result, the statistical analysis is interpreted as supporting evidence of consistency of performance, not as definitive evidence that independent samples are generalizable.

We used these subsets for five repeated evaluation runs, enabling us to measure performance statistically across multiple iterations. The proposed hybrid model, which combines a KNN classifier with bi-lightweight KD-directed CNNs (guided by DenseNet121 and MobileNetV2), was statistically compared to its teacher models, DenseNet121 and MobileNetV2. The evaluation framework is to determine whether the performance gains of the hybrid strategy over the teacher models are statistically significant and not the result of chance.

This statistical analysis was carried out for five repeated evaluation runs to measure the performance of the proposed Hybrid Approach-Bi-lightweight KD-guided CNNs guided by DenseNet121 and MobileNetV2 with a KNN classifier, as shown in Table 14. The proposed Hybrid approach provided a better F1-Score and lower FPR and FNR values than both teachers. On average, the F1-Score value for the Hybrid approach was 95.90% ± 0.22 compared to 93.49% ± 0.30 for DenseNet121 and 92.99% ± 0.25 for MobileNetV2. Also, the FPR (4.53%) and FNR (3.77%) values were better for the Hybrid approach than for the corresponding teacher models. The 95% confidence intervals (CIs) estimated on the basis of the t-distribution during five repeated runs further demonstrate consistent performance across the evaluation runs. Finally, the results of the paired t-test reveal statistically significant differences between the Hybrid approach and both teachers. After carrying out the Bonferroni correction for six pairwise comparisons (adjusted *α* = 0.0083), all six pairwise comparisons were statistically significant, with adjusted *p*-values varying between 5.99E-6 and 3.76E-3. All of these statistics confirm consistent performance of the proposed Hybrid approach better than the performance of two teacher models. However, these statistics have to be considered as the results of repeated evaluation runs rather than as a replacement for independent external/clinical validation.

**Table 14:** Comparison of statistical analysis outcomes conducted among the proposed hybrid approach and corresponding teacher models.

Approach		Proposed hybrid Approach			Densenet121 (Teacher Model)			MobileNetV2 (Teacher Model)		
		FS (%)	FPR (%)	FNR (%)	FS (%)	FPR (%)	FNR (%)	FS (%)	FPR (%)	FNR (%)
Metrics Attained in Five Individual Test Runs	Run1-Set1	95.68	4.81	3.97	93.34	6.54	5.95	92.7	7	5.92
	Run2-Set2	95.97	4.69	4.15	93.45	6.65	5.91	92.87	7.11	6.05
	Run3-Set3	95.67	4.47	3.85	93.11	6.38	5.75	92.9	6.96	6.28
	Run4-Set4	96.04	4.45	3.35	93.72	6.06	5.34	93.2	6.65	6.42
	Run5-Set5	96.15	4.24	3.52	93.85	6.15	5.45	93.3	6.76	6.58
Mean ± Std. Dev		95.90±0.22	4.53±0.22	3.77±0.33	93.49±0.30	6.36±0.25	5.68±0.27	92.99±0.25	6.90±0.19	6.25±0.27
Mean ± 95%CI		95.90±0.27	4.53±0.28	3.77±0.41	93.49±0.37	6.36±0.31	5.68±0.34	92.99±0.31	6.90±0.23	6.25±0.33
Paired t-test								FS	FPR	FNR
Bonferroni-adjusted *p*-value for Proposed Hybrid Approach vs. Densenet121 (Teacher Model)								9.48E-6	6.18E-5	7.86E-6
Bonferroni-adjusted *p*-value for Proposed Hybrid Approach vs. MobileNetV2 (Teacher Model)								5.99E-6	2.89E-5	3.76E-3
*Proposed Approach: Hybrid Approach with Bi-lightweight KD-guided CNN Models (Guided by Densenet121 and MobileNetV2) with KNN Classifier*										

### 5.8 Explainability analysis

To ensure the explainability and interpretability of the proposed hybrid architecture, an explainability analysis is done for both the CNN-based feature extractors and the Machine Learning (ML) classifiers. For the explainability analysis, the better-performing hybrid architecture of the Bi-lightweight KD-guided CNN feature extractors (with DenseNet121 and MobileNetV2 teacher networks) and the KNN classifier is selected. Grad-CAM visualization, as well as DAUC (Deletion Area Under Curve) and IAUC (Insertion Area Under Curve) measures, are used to see whether the Bi-lightweight KD-guided CNN architectures emphasize sickle cell parts of the image during prediction or not. Besides, SHAP (SHapley Additive exPlanation) is used to determine the role of the features obtained from CNN architectures in the decisions taken by the ML classifiers.

Figure 16 shows the GradCAM visualizations for the student models guided by the DenseNet121 and MobileNetV2 teacher models, respectively. Despite the similar architecture of these two students, their teachers are different, implying the acquisition of distinct feature representations during training. In particular, both student models detect abnormal regions with red blood cells in the diseased sample, whereas their activations in the healthy sample are much weaker and more diffuse. The distinctive attention patterns reveal that each teacher network imparts unique discriminative knowledge to its students. This explainability qualitative assessment can be further confirmed by the numerical results presented in Table 15 and Figure 17, which show that both student models exhibit consistently high IAUC values (0.734–0.785) and relatively low DAUC values (0.426–0.486). High IAUC values mean that the highlighted regions contain informative features that significantly increase prediction confidence, while relatively low DAUC values confirm that excluding these regions from consideration causes a decrease in confidence level. As there are several sickled cells in one image, the confidence reduces gradually, not drastically, which is reflected in the moderate DAUC values.

**Figure 16:**
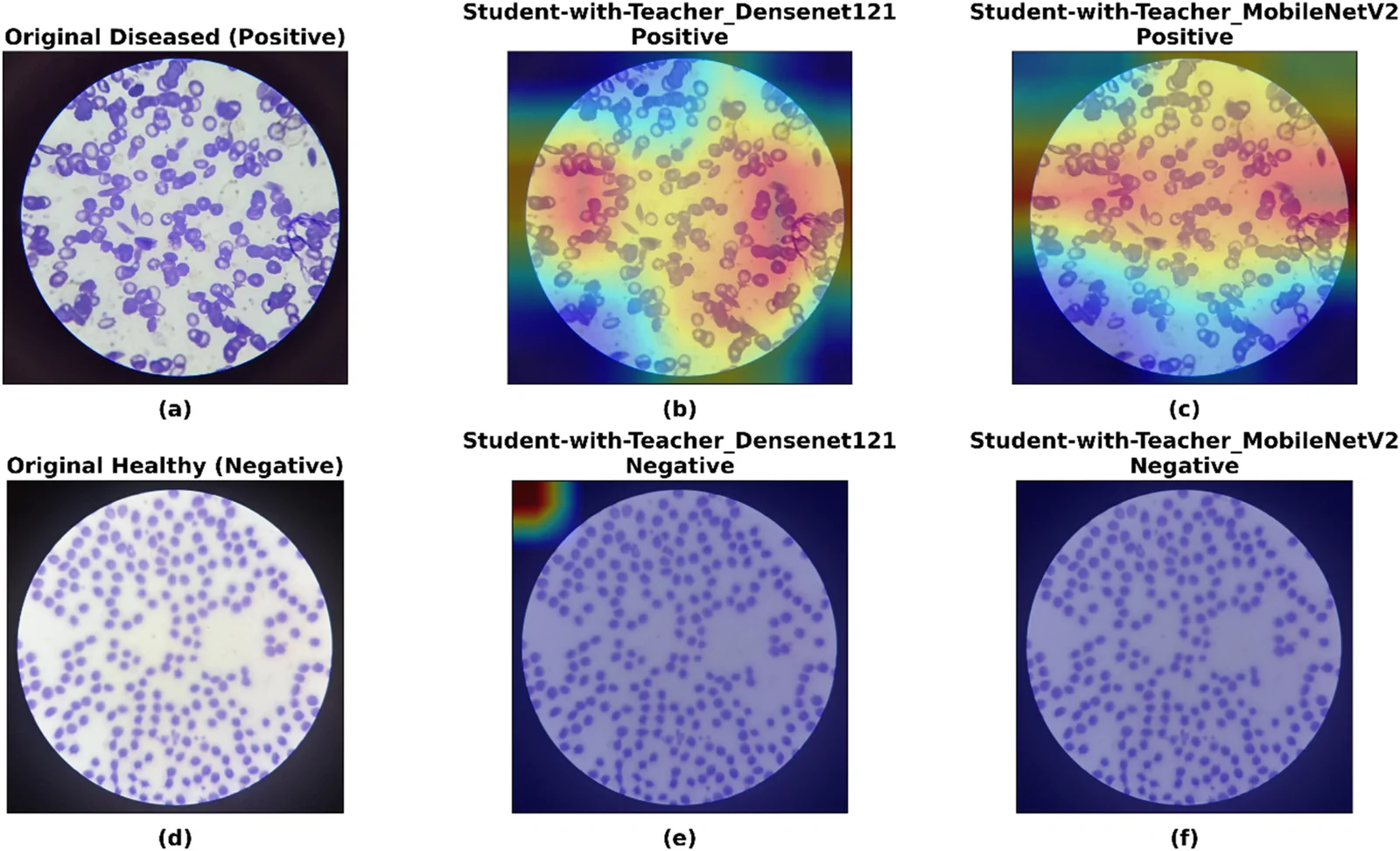
Visualization of GradCAM generated by the two individual student models distilled by the two different teacher models (DenseNet121 and MobileNetV2) for sample images. **(a,d)** Original images of Diseased (Positive) and Healthy (Negative) samples, **(b)** GradCAM for diseased (positive) generated by the student-with-teacher DenseNet121, **(c)** GradCAM for diseased (positive) generated by the student-with-teacher MobileNetV2, **(e)** GradCAM for healthy (negative) generated by the student-with-teacher DenseNet121, **(f)** GradCAM for healthy (negative) generated by the student-with-teacher MobileNetV2.

**Table 15:** Outcome of the quantitative evaluation of GradCAM images generated by the two feature extractors present in the Bi-lightweight KD-guided CNN feature extractors (guided by DenseNet121 and MobileNetV2 teacher networks) and the KNN classifier hybrid model.

Feature extractor	Class	IAUC	DAUC
Student-with-Teacher DenseNet121	Diseased (Positive)	0.781	0.426
	Healthy (Negative)	0.734	0.486
Student-with-Teacher MobileNetV2	Diseased (Positive)	0.785	0.432
	Healthy (Negative)	0.742	0.475

**Figure 17:**
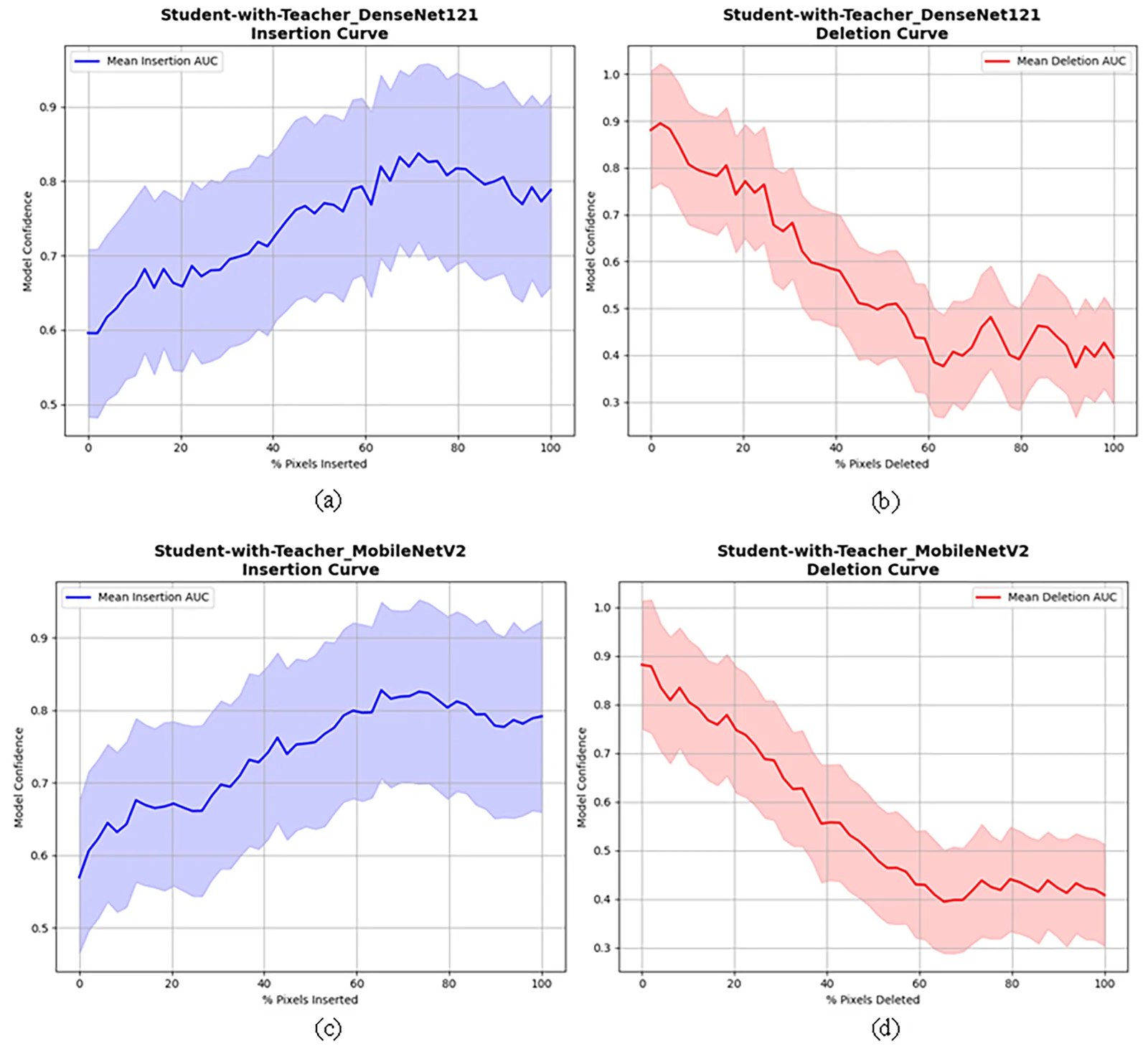
Visualization of IAUC and DAUC curves of the two individual student models distilled by the two different teacher models (DenseNet121 and MobileNetV2). **(a)** IAUC curves of the Student-with-Teacher DenseNet121, **(b)** DAUC curves of the Student-with-Teacher DenseNet121, **(c)** IAUC curves of the Student-with-Teacher MobileNetV2, **(d)** DAUC curves of the Student-with-Teacher MobileNetV2.

Figure 18 is the output from SHAP analysis for the KNN classifier incorporated with Bi-lightweight KD-guided CNN models. As seen from Figure 18, the classifier uses the features from both of the teacher-guided student models, such as DenseNet121-guided and MobileNetV2-guided models, and there is a unique set of high-impact features for predicting diseases. The existence of high-impact features from both models implies that each student model contributes uniquely to the model output. Therefore, using the features extracted from both models leads to the creation of an informative representation space, which leads to better disease classification performance.

**Figure 18:**
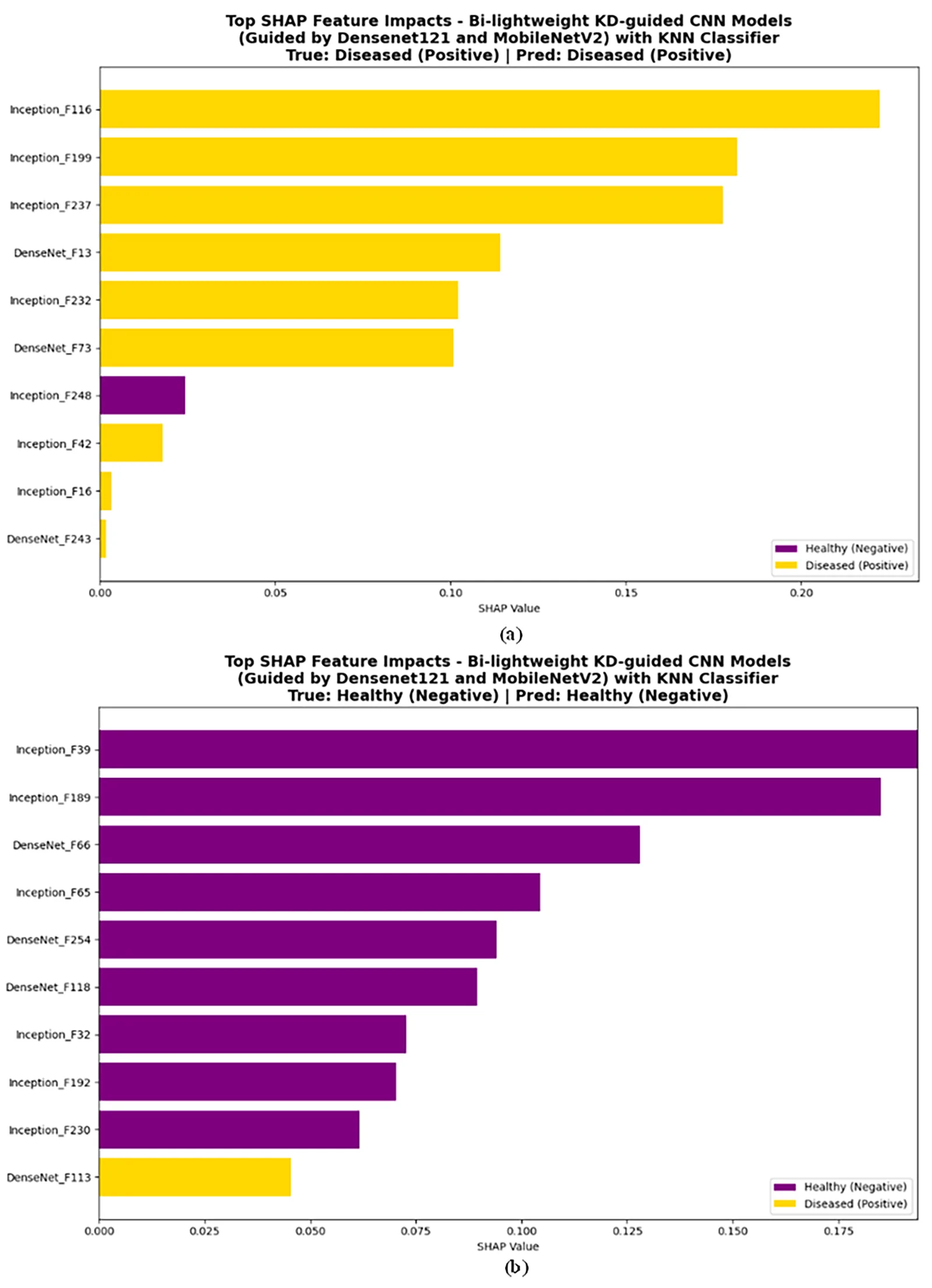
Visualization of the SHAP plot of the KNN classifier used in the Bi-lightweight KD-guided CNN models (guided by Densenet121 and MobileNetV2) with KNN classifier for the sample image. **(a)** SHAP plot for the Diseased (Positive) sample, **(b)** SHAP plot for the Healthy (Negative) sample.

However, SHAP analysis does not replace clinical validation or expert assessment, even though it provides insight into the features contributing to model predictions. Further testing of the diagnostic reliability of the proposed framework will require the use of clinically representative datasets and comparisons with expert diagnoses made by hematologists/pathologists.

### 5.9 Ablation study

Comprehensive ablation research is conducted to evaluate every architectural choice and its impact on the overall performance and efficiency of the model. The ablation study is conducted on the following aspects.

- Selection of Transfer Learning Configuration
- Selection of Global Average Pooling (GAP) and Fully Connected (FC) layers in the classification head
- Selection of Optimizer and Learning_rate
- Selection of T value in Knowledge Distillation
- Selection of Quantization techniques used in TFLite conversion
- Evaluation of Model Performance on Original and Augmented Test Sets
- Evaluation of Model Generalizability Using k-Fold Cross-Validation
- Comparison of Bi-lightweight and Ensemble-Teacher Knowledge Distillation Strategies

#### 5.9.1 Selection of transfer learning configuration

The impact of freezing backbone layers on teacher model accuracy and training efficiency is initially evaluated by comparing **Trainable = True** vs. **Trainable = False** settings in an ablation study on a transfer learning setup. This is essential to the success of Knowledge Distillation because trained teacher models with refined feature representations may offer more informative soft targets, which help the student model learn and generalize more successfully.

The results of an ablation study to identify the best transfer learning configuration—Trainable = True vs. Trainable = False, for constructing the teacher model in the proposed knowledge distillation framework, are shown in Table 16. To ensure a fair comparison, all candidate models, including MobileNetV2, InceptionV3, DenseNet121, and ResNet50, are trained for 25 epochs under the same settings.

**Table 16:** Outcome of the ablation study on the test dataset to select a suitable configuration for transfer learning to build the teacher model.

S.No	Transfer learning configuration	Teacher model	Accuracy	F1-score
			(%)	%
1	True	MobileNetV2	93.74	93.07
2	False	MobileNetV2	87.22	86.62
3	True	InceptionV3	91.33	90.22
4	False	InceptionV3	84.44	83.81
5	True	Densenet121	94.21	93.57
6	False	Densenet121	87.97	87.41
7	True	ResNet50	92.05	91.45
8	False	ResNet50	85.3	84.71

Making the base network trainable consistently outperformed the frozen (non-trainable) configuration in Accuracy and F1-score across all architectures. Because it enables the model to modify its pre-trained weights to the specific domain of sickle cell identification, this highlights the significance of fine-tuning. Improved representation learning, better class discrimination, and improved overall performance, especially in detecting small morphological variations, are the results of the model's increased ability to collect domain-specific data. The study demonstrates that improving the teacher model's feature quality by base layer fine-tuning enhances knowledge transfer to student models.

#### 5.9.2 Selection of global average pooling (GAP) and fully connected (FC) layers in classification head

The essential transition between the dense classification block and the deep convolutional layers in the proposed lightweight student model is Layer 15 (Table 3). We currently examine two possible configurations: a Fully Connected (Dense) layer and GlobalAveragePooling2D. An ablation study is carried out to select architectural elements that guarantee the optimal balance between accuracy, model complexity, and real-time deployability in resource-constrained environments in order to identify the best design in terms of classification performance and computational efficiency.

An ablation study assessing the impact of various Layer 15 configurations—Global Average Pooling (GAP) and Fully Connected (FC) layers with 1024, 512, and 256 neurons—on the complexity and performance of the proposed student model without KD is shown in Table 17. With zero trainable parameters at Layer 15, the GAP layer provides the lowest complexity (0.65M parameters, 6.47 MB) with 63.91% accuracy and 62.10% F1-score, making it appropriate for applications with limited memory and power.

**Table 17:** Outcome of the ablation study on the test dataset to select a suitable configuration for layer 15 in the classification head of the proposed lightweight student model.

Model name	Layer 15 configuration	Test dataset			
		NCP	Model size	Accuracy	F1-score
		Million	MB	(%)	(%)
Proposed Student Model with Different Layer 15 Choices	GAP Layer	0.65	6.47	63.91	62.10
	FC Layer (1024)	1.30	12.95	65.42	64.71
	FC Layer (512)	0.91	9.10	64.13	63.45
	FC Layer (256)	0.713	7.10	61.19	60.46

NCP, number of parameters generated by the model; GAP, global average pooling; FC, fully connected.

Trainable parameters are increased based on the number of neurons by adding fully connected layers at Layer 15: FC-1024 adds 525,312, FC-512 adds 262,656, and FC-256 adds 131,328. The size and complexity of the model are significantly impacted by these additions. Although the model size is nearly doubled to 12.95 MB (1.30M parameters) in the FC-1024 configuration, which achieves 65.42% accuracy and 64.71% F1-score, the computational cost is greater than the slight performance increase over GAP. At 9.10 MB (0.91M parameters), the FC-512 model offers a better trade-off with 64.13% Accuracy and a 63.45% F1-score. Nevertheless, it still adds a significant amount of computational complexity in comparison to GAP, which needs to be taken into account in situations when resources are limited. However, while being lighter (7.10 MB, 0.713M parameters), FC-256 performs the less, falling short of the GAP baseline with an accuracy of 61.19% and an F1-score of 60.46%. Thus, as demonstrated by the experimental findings in Table 16, the GAP design consistently strikes a good compromise between accuracy and efficiency, confirming its applicability for real-time clinical applications on devices with limited resources.

#### 5.9.3 Selection of optimizer and learning_rate

The main objective of this analysis is to assess how various optimizers and learning rates affect the convergence and generalization capabilities of both teacher and student models. Based on previous results (as indicated in Table 17), we choose DenseNet121 and MobileNetV2, the two best-performing teacher models, as representative teacher models. For consistency and control, we employ the proposed lightweight Student-without-KD model (Table 6). To isolate the direct effects of optimizer and learning rate selections, the student model is carefully chosen to provide a baseline for comparing optimization strategies without the additional influence of knowledge distillation. To align with the initial training setup, the student model is trained for 100 epochs and the teacher model for 25 epochs.

The three popular optimizers, such as ADAM, RMSprop, and SGD—as well as two learning rates (0.001 and 0.0001) are taken into consideration in this ablation study experiment since they are effective in existing studies (45, 46). The corresponding outcomes on the test dataset with these different configurations are shown in Table 18.

**Table 18:** Outcome of the ablation study on the test dataset to select a suitable Optimizer with Learning_rate.

Models	Optimizer	Learning_rate = 0.001		Learning_rate = 0.0001	
		Accuracy	F1-score	Accuracy	F1-score
		(%)	(%)	(%)	(%)
DenseNet121	ADAM	94.21	93.57	93.69	93.36
	RMSProp	92.49	92.11	91.56	91.10
	SGD	91.29	90.81	89.81	89.28
MobileNetV2	ADAM	93.74	93.07	93.20	92.86
	RMSProp	91.85	91.41	90.86	90.41
	SGD	90.53	90.05	88.93	88.39
Student-without-KD model	ADAM	63.91	62.10	63.20	62.44
	RMSProp	61.83	61.13	60.62	59.93
	SGD	60.38	59.61	58.51	57.76

In terms of accuracy and F1-score, the ADAM optimizer consistently performs better than both RMSProp and SGD across all models and configurations, independent of learning rate. With a learning rate of 0.001, for example, ADAM outperforms SGD with significantly lower metrics (91.29%/90.81%), but ADAM performs best with 94.21% accuracy and 93.57% F1-score in the case of DenseNet121. A similar pattern is shown with MobileNetV2, where ADAM once more produces the best outcomes, demonstrating its resilience when dealing with intricate feature representations during fine-tuning.

Even though RMSProp produces relatively competitive results, it continuously performs lower than ADAM. The SGD optimizer performs poorly in this scenario, which is most likely because of the short training period (25 epochs), where its slower convergence reduces efficacy. With the highest accuracy (63.91%) and F1-score (62.10%) of all studied optimizers, ADAM exhibits superior performance in the Student-without-KD model. Overall, the findings show that ADAM, with a learning rate of 0.001, is the most well-balanced and effective arrangement for the study's Teacher and Student models.

#### 5.9.4 Selection of T value in knowledge distillation

Temperature (T) regulates the softness of the teacher model's output in the sickle cell detection knowledge distillation process, which is essential for conveying beneficial inter-class relationships, particularly when identifying minute morphological variations. The student model can learn both the proper class and class similarities with the help of softer outputs (higher T values), which improves generalization. Low T values, on the other hand, result in forecasts that are too confident, which restricts the transfer of knowledge, while high T values might make it difficult to distinguish between classes. Consequently, T tuning is crucial. Because they provide a fair trade-off between class clarity and information depth, T values of 2, 3, and 4 are investigated in this study. T = 4 enables more nuanced feature learning, which aids in determining the ideal setting; T = 3 facilitates optimal generalization, and T = 2 offers mild softening.

We chose Student-with-Teacher_DenseNet121 and Student-with-Teacher_MobileNet V2 for this investigation since they outperformed other variations and the two top-performing KD-guided student models according to Table 6. Within the framework of knowledge distillation, these models are assessed using varying temperature (T) values of 2, 3, and 4, directed by their respective teacher networks, DenseNet121 and MobileNetV2. The ablation study results are summarized in Table 19, which also shows the effect of these temperature changes on sickle cell classification performance.

**Table 19:** Outcome of the ablation study on the test dataset to select the T value to be used in knowledge distillation.

Models	T = 2		T = 3		T = 4	
	Accuracy	F1-score	Accuracy	F1-score	Accuracy	F1-score
	(%)	(%)	(%)	(%)	(%)	(%)
Student-with-Teacher_MobileNetV2	84.72	84.30	85.35	83.50	84.92	84.30
Student-with-Teacher_Densenet121	85.25	84.51	85.89	84.20	85.11	84.51

According to Table 19, T = 3 consistently yields the maximum accuracy (85.35%, 85.89%) and F1-score (83.50%, 84.20%) for both student models—Student-with-Teacher_MobileNetV2 and Student-with-Teacher_DenseNet121—achieving the best possible balance between class discriminability and softer supervision. Although T = 2 and T = 4 produce competitive outcomes, they perform marginally worse: T = 4 may over-smooth the teacher's output, blurring class boundaries, while T = 2 delivers little inter-class knowledge. The resilience of the KD process is demonstrated by the limited fluctuation (<1%) across T values, which validates that well-regularized student models successfully adjust to varying degrees of softened targets. According to these findings, T = 3 is the best option for distillation applications requiring subtle morphological distinction, like sickle cell detection. Hence, the hybrid approach that combines an ML classifier with a bi-lightweight KD-guided CNN moves on with this setup.

#### 5.9.5 Selection of quantization techniques used in TFLite conversion

Three different TFLite quantization approaches are used in an ablation study to assess the deployment effectiveness of the proposed student models on devices with limited resources: Float32, Float16 (FP16), and INT8. To determine their effect on classification performance and computing efficiency, these quantized models are incorporated into the proposed hybrid framework, which combines a Bi-lightweight KD-guided CNN with an ML classifier. While FP16 provides a balanced reduction in model size with low accuracy loss, Float32 retains full precision with only modest gains in size or latency. The fastest inference and maximum compression are offered by INT8, while performance may be marginally lowered based on calibration. Table 19 shows the Accuracy, F1-Score, Model Size, and MIT used to evaluate every configuration. Here, the MIT is calculated in the CPU environment in the absence of a GPU and an edge device like the Raspberry Pi Model B. The choice of the best quantization technique for edge-based, real-time sickle cell identification is supported by this investigation.

Table 20 shows that the highest classification performance is achieved by Float32-quantized models, with 95.45% accuracy and a 92.46% F1-Score. In comparison, FP16 and INT8 show a modest 2%–4% drop in performance due to rounding errors and quantization sensitivity, respectively. However, both significantly reduce the model size (FP32: 3.96 MB → FP16: 2.01 MB; INT8: 1.20 MB) and improve inference time, particularly on the Raspberry Pi. On this edge device, the Mean Inference Time (MIT) reduces from 206 ms (FP32) to 185 ms (FP16) and 162 ms (INT8), enabling faster execution for real-time applications. These gains are more pronounced on Raspberry Pi compared to CPU environments, highlighting the benefit of quantization for resource-constrained deployments. In applications such as sickle cell identification, even small performance declines might result in clinically significant mistakes, even with the efficiency gains. Even though FP32 requires a little more computing, it is still controllable on Raspberry Pi devices and is warranted given the possible loss of diagnostic precision. FP32 is chosen for this study's TFLite conversion because it provides the most dependable trade-off between clinical performance and computational expense.

**Table 20:** Outcome of the ablation study on the Test dataset to select the quantization technique to be used in the TFLite conversion process.

Evaluation Metrics		Proposed hybrid Approach WITH FP32 quantized student models and KNN classifier	Proposed hybrid approach with FP16 quantized student models and KNN classifier	Proposed hybrid approach with INT8 quantized student models and KNN classifier
Accuracy	(%)	95.45	93.24	91.62
F1-score	(%)	92.46	90.04	88.96
Model size	(MB)	3.96	2.011	1.20
MIT in CPU	(ms)	113	110	103
MIT in raspberry Pi	(ms)	206	185	162

#### 5.9.6 Evaluation of model performance on original and augmented test sets

To investigate the effect of test-set augmentation on the obtained results, the best-performing two hybrid configurations of the framework, which are Bi-lightweight KD-guided CNNs based on DenseNet121 and MobileNetV2 with KNN and SVM classifiers respectively, were chosen to perform comparative analysis. Both configurations were evaluated using the augmented test set and the original unaugmented test set. Augmented-set evaluation demonstrates the robustness of the framework under image variation conditions; on the other hand, original test-set evaluation gives a more direct insight into the generalization capability of the framework. This comparison allows determining the consistency of the framework performance on the two sets of conditions.

Table 21 contains the comparative results of the performance of the selected hybrid models on the augmented test dataset and on the original test dataset. The KNN-based hybrid model showed 95.45% accuracy and 95.02% F1-score on the augmented test dataset, which slightly increased to 95.95% accuracy and 95.58% F1-score on the original test dataset. Likewise, the SVM-based hybrid model increased its performance from 93.68% to 94.11% accuracy and from 93.28% to 93.73% F1-score on the original test dataset.

**Table 21:** Performance analysis of selected hybrid models under augmented and original test datasets.

Model	Model format	Testing time performance metrics on augmented dataset		Testing time performance metrics on un-augmented actual dataset	
		A	FS	A	FS
		(%)	(%)	(%)	(%)
Hybrid Approach with Bi-lightweight KD-guided CNN Models (Guided by Densenet121 and MobileNetV2) with KNN Classifier	.tflite	95.45	95.02	95.95	95.58
Hybrid Approach with Bi-lightweight KD-guided CNN Models (Guided by Densenet121 and MobileNetV2) with SVM Classifier	.tflite	93.68	93.28	94.11	93.73

The slight improvement in performance on the original test dataset may be due to controlled variations applied during test-set augmentation, which can marginally affect the classification process. Since the training datasets were augmented before the model training, the augmentation technique was consistently applied to increase training diversity, while the original test dataset was kept unchanged to additionally evaluate model performance on held-out samples. The small difference between the two evaluation techniques suggests that the reported performance is not significantly affected by test-set augmentation. Nevertheless, since both test datasets are generated from the same dataset, evaluation of the model on multi-source independent datasets is required, which will be conducted in future work.

#### 5.9.7 Evaluation of model generalizability using k-fold cross-validation

For further evaluation of the better-performing and balanced proposed hybrid model in terms of robustness and generalizability, 5-fold cross-validation was carried out as an additional ablation analysis. The experiments took into account the Bi-lightweight KD-guided CNN models, guided by DenseNet121 and MobileNetV2, with a KNN classifier. To prevent the possibility of data leakage, initially, the original images were separated into five folds before the data augmentation was applied. The validation fold was left untouched and remained in its initial form, whereas the augmentation procedure was applied to the training folds and increased their size up to 500 images per class. For all five folds, the Accuracy and F1-score values were recorded and summarized using mean ± standard deviation. Using the splitting-first augmentation technique, one can be sure that there will be no augmented version of the same original image in the training and validation folds.

The 5-fold cross-validation results of the proposed Bi-lightweight KD-guided hybrid model, which uses the DenseNet121- and MobileNetV2-guided student models with a KNN classifier, are shown in Table 22. The model shows uniform performance across all five folds, as Accuracy varied between 95.40% and 95.46%, while the F1-score changed between 94.89% and 95.05%. The overall performance of 95.43 ± 0.03% Accuracy and 94.98 ± 0.07% F1-score proves the lack of variability across different data splits. The split-first augmentation technique guarantees that the validation fold remains untouched and no augmented version of the same original images appears in the training and validation sets.

**Table 22:** Performance of the proposed Bi-lightweight KD-guided hybrid model under 5-fold cross-validation with split-first data augmentation.

Model		Testing Time performance metrics on augmented dataset	
		A	FS
		(%)	(%)
Hybrid Approach with Bi-lightweight KD-guided CNN Models (Guided by Densenet121 and MobileNetV2) with KNN Classifier	Fold 1	95.46	95.05
	Fold 2	95.45	95.03
	Fold 3	95.44	95.01
	Fold 4	95.41	94.91
	Fold 5	95.40	94.89
	Mean + Std	95.43 ± 0.03	94.98±0.07

#### 5.9.8 Comparison of Bi-lightweight and ensemble-teacher knowledge distillation strategies

To support this proposed Bi-lightweight KD-guided hybrid architecture, an ablation experiment is conducted by comparing the architecture against a more straightforward single-student ensemble-teacher knowledge-distillation method. In this alternative setup, the outputs of the DenseNet121 and MobileNetV2 teachers are averaged through a weighted-averaging mechanism to create an ensemble-teacher signal. Weighted averaging was intentionally used here as it represents a very efficient mechanism that preserves soft probability distributions (dark knowledge) necessary for distillation. This aggregated knowledge is used to train the single lightweight student model. Later, the extracted features from this student model are used with the KNN classifier to create the corresponding hybrid model.

From Table 23, the proposed Bi-lightweight architecture achieves 95.45% Accuracy and 95.02% F1-score, while the single-student ensemble-teacher architecture achieves 93.29% Accuracy and 92.85% F1-score on the augmented test dataset. Thus, in this case, the observed improvement equals 2.16% for Accuracy and 2.17% for F1-score, correspondingly.

**Table 23:** Performance comparison of the Bi-lightweight KD-guided hybrid model and the single-student ensemble-teacher KD-based hybrid model.

Model	Testing time performance metrics	
	A	FS
	(%)	(%)
Hybrid Approach with Bi-lightweight KD-guided CNN Models (Guided by Densenet121 and MobileNetV2) with KNN Classifier	95.45	95.02
Hybrid Approach with Single KD-guided CNN Model – (Guided by Ensembled Teachers Densenet121 and MobileNetV2) with KNN Classifier	93.29	92.85

This observed improvement suggests that, under the conditions of the current experimental setup, the independent distillation of DenseNet121 and MobileNetV2 knowledge into two student branches is much better than consolidating the teacher knowledge before distillation. It should be noted that such a performance gap is due to the prevention of the smoothing process of features. Indeed, since DenseNet121 and MobileNetV2 use completely different architectural paradigms, the consolidation of their outputs through weighted averaging into a single supervisory signal may lead to the smoothing of the sharp and unique decision boundaries obtained by each teacher, which limits the single student model. On the contrary, in the Bi-lightweight approach, each student branch manages to preserve the unique representation space of its teacher. Then, when concatenated, these unique feature spaces provide the downstream KNN classifier with a richer feature matrix.

Nevertheless, it is important to emphasize that these results should be interpreted specifically as the empirical comparison with the single-student ensemble-teacher strategy, not as proof of the general superiority over all multi-teacher distillation approaches. Hence, this ablation study serves as the solid architectural justification of the proposed Bi-lightweight dual-branch design within the framework of this experiment.

## 6 Conclusion

This work implemented a hybrid framework that combines machine learning and computer vision methods with the Knowledge Distillation process for precise and effective sickle cell detection. The method combined conventional classifiers with bi-lightweight KD-Guided lightweight CNN models to strike a balance between computational efficiency and diagnostic performance. For training and validation, the Sickle Cell Disease Dataset is utilized.

Knowledge distillation (KD) is at the core of the proposed approach, wherein outstanding teacher models like DenseNet121, MobileNetV2, ResNet50, and InceptionV3 supervise the training of lightweight student models built using depthwise separable convolution layers. Without adding to the computational cost, this method allows student models to inherit the representational capabilities of deeper designs. Among these, student models with DenseNet121 and MobileNetV2 guidance showed significant improvements in diagnosis. Along with notable decreases in loss (22.72%), FPR (16.25%), and FNR (28.13%), the DenseNet121-guided model outperformed the non-KD student model by increasing accuracy by 21.98% and F1-score by 22.10%. Comparable gains are also seen by the MobileNetV2-guided student model, which saw a significant decrease in loss, an increase in the F1-Score of 21.4%, and an accuracy gain of over 21.44%. These results demonstrate how well hierarchical knowledge transfer works to enhance lightweight sickle cell detection models.

The outputs of the two best-performing KD-guided student models are merged in a hybrid ensemble with a K-Nearest Neighbors (KNN) classifier to further improve prediction accuracy and generalization. The decision boundary benefits of traditional machine learning classifiers and the rich feature representations from KD-guided CNN models are both successfully captured by this combination. Clinical-grade performance depends on the dual-model integration's ability to handle the minute morphological distinctions between sickle and normal red blood cells.

The KD-guided models are transformed to TensorFlow Lite (TFLite) using Float32 quantization for deployment on devices with limited resources. Achieving a mean inference time of 113 ms and reducing the hybrid model size to 3.96 MB allowed for an acceptable precision (95.45%) and F1-score (92.46%). A lightweight architecture and real-time performance demonstrated on the Raspberry Pi 4 Model B enable the framework to support edge-based and point-of-care applications in resource-constrained environments. The clinical applicability of the model still needs to be validated using larger and multisource datasets as well as expert-assisted clinical evaluations.

### 6.1 Limitations and future work

- This research depends on one publicly accessible database, which has only a few samples, meaning it may not be able to provide a full representation of the variance found in clinical practice. Even though data augmentation was applied to increase data diversity and statistical significance analysis was conducted on an augmented test database to improve validation accuracy, the analysis still relies on a single data source. Future work will investigate the validity of the proposed method using larger, more balanced, and multi-source datasets to establish its generalizability and clinical applicability further.
- Despite promising performance, successful Raspberry Pi deployment, and explainable predictions using GradCAM and SHAP analyses, the proposed framework has not been validated in a real clinical setting or compared with expert hematologist/pathologist diagnoses. Future work will focus on clinical validation and expert-assisted evaluations, which will enable us to further establish its diagnostic reliability and clinical applicability.
- The statistical analysis was done through augmented data from a small pool of original test images. This may mean that the dependencies within the augmented variations would impact the assumption of independence for the statistical tests. In future work, we will be using larger independent datasets and a controlled statistical design to prove the generalizability of the framework.
- In this research work, the testing of the framework is done using a balanced test dataset, while the Positive Predictive Value (PPV) is affected by the prevalence of disease. This means that the Accuracy and the F1-scores obtained in this research work do not represent the PPV in the real world, where there is a reduced prevalence of sickle-cell disease. In future work, the framework will be tested on clinically realistic datasets, and PPV, Negative Predictive Value (NPV), and other prevalence-based metrics will be provided.
- Despite the performance-complexity trade-off provided by the Bi-lightweight framework, the ablation analysis is limited to a single-student ensemble-teacher strategy based on weighted averaging of DenseNet121 and MobileNetV2 outputs. Future work will continue to examine various multi-teacher KD strategy mechanisms.

## Data Availability

The data that support the findings of this study are openly available in the Sickle Cell Disease Dataset at https://www.kaggle.com/datasets/florencetushabe/sickle-cell-disease-dataset.
